# Mesoporous N,S‐Dual‐Doped Carbon Nanoreactors via Entropy‐Driven Interface Self‐Assembly for Efficient H_2_O_2_ Electrosynthesis

**DOI:** 10.1002/anie.7636911

**Published:** 2026-05-23

**Authors:** Fei Liu, Xiaoqing Liu, Rui Zhang, Linxia Cui, Ji Liang, Rui Gao, Jian Liu

**Affiliations:** ^1^ College of Chemistry and Chemical Engineering Inner Mongolia Key Laboratory of Rare Earth Catalysis Inner Mongolia University Hohhot China; ^2^ Key Laboratory of Advanced Ceramics and Machining Technology Ministry of Education Tianjin University Tianjin China; ^3^ Institute For Green Chemistry and Environmental Science Inner Mongolia University Hohhot China; ^4^ DICP‐Surrey Joint Centre For Future Materials Department of Chemical and Process Engineering and Advanced Technology Institute University of Surrey Guildford UK

**Keywords:** entropy‐driven self‐assembly, heteroatom doping, hydrogen peroxide, mesoporous carbon, oxygen reduction reaction

## Abstract

The electrochemical two‐electron oxygen reduction reaction (2e^−^ ORR) offers a sustainable route for H_2_O_2_ production. Rational catalyst design is essential for achieving efficient H_2_O_2_ electrosynthesis, in which porous heteroatom‐doped carbon‐based materials hold tremendous potential. Nevertheless, the simultaneous realization of homogenized heteroatom doping and a precisely engineered porous structure in the carbon skeleton remains a significant challenge. Herein, we propose an entropy‐driven interface self‐assembly strategy to fabricate mesoporous N,S‐dual‐doped carbon‐based nanoreactors with tunable geometries. The optimal sample shows exceptional performance in a flow cell, achieving H_2_O_2_ production rate of 17.38 mol_gcat_^−1^ h^−1^ at −0.2 V versus reversible hydrogen electrode (RHE) with > 90% selectivity. DFT calculations and finite element analysis simulations reveal that the N,S‐dual‐doping configuration optimizes the *OOH adsorption energy, while the well‑defined mesoporous structure accelerates mass transport and promotes the enrichment of surface O_2_ concentration. This work provides a general principle for synergizing heteroatom doping and nanostructural engineering toward high‐performance electrocatalysts for sustainable synthesis.

## Graphical abstract

An entropy‐driven interface self‐assembly strategy is proposed for the controllable synthesis of mesoporous N,S‐dual‐doped carbon nanoreactors. The N,S‐dual‐doping sites optimized the *OOH adsorption, boosting the 2e^−^ selectivity, while the favorable mesostructure accelerated O_2_ enrichment. These factors collectively contributed to an enhanced overall O_2_‐to‐H_2_O_2_ conversion efficiency in water.

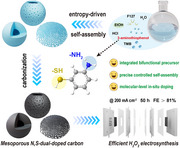

## 1 Introduction

Hydrogen peroxide (H_2_O_2_), one of the 100 most important chemicals in the world, serving as a potent, environmentally benign, and atom‐economical oxidant, is widely applied in various fields, such as fine chemicals, the disinfection of drinking water, paper bleaching, and wastewater treatment [1, 2, 3]. Recently, compared to the well‐established energy‐intensive anthraquinone process, the electrochemical two‐electron oxygen reduction reaction (2e^−^ ORR), as a low‐cost, safe, and continuous alternative route for the H_2_O_2_ production [4, 5, 6, 7, 8, 9, 10], plays a pivotal role in promoting chemical industry and energy system towards a green, sustainable, and carbon‐neutral direction.

Currently, the prerequisite for efficient electrosynthesis of H_2_O_2_ is developing appropriate electrocatalysts to enhance and optimize the activity and selectivity of 2e^−^ ORR. Among the reported electrocatalysts, metal‐free carbon‐based nanocatalysts have become a promising candidate for H_2_O_2_ electrosynthesis due to their low cost, superior electrochemical conductivity, enriched surface chemistry, and variable morphology [11, 12, 13, 14, 15]. Nevertheless, it is still a tremendous challenge to obtain preeminent activity and H_2_O_2_ selectivity over a wide pH range based on 2e^−^ ORR carbon‐based catalysts. Because of the weak thermodynamic competitiveness of H_2_O_2_ in 2e^−^ ORR [16, 17], the four‐electron oxygen reduction reaction (4e^−^ ORR) pathway seriously destroys the yield of H_2_O_2_. In recent years, numerous nanochemical synthesis strategies, such as defect design [18], heteroatom‐doping [19, 20, 21], interfacial functionalization modulation [22, 23], and so forth, have been exploited to effectively improve 2e^−^ selectivity at the atomic level and nanoscale. Meanwhile, the generated H_2_O_2_ in situ tends to accumulate in the catalyst layer, leading to further electric reduction [24, 25]. Studies have indicated that accelerating the local diffusion rate and promoting the rapid escape of H_2_O_2_ are conducive to the positive shift of reaction equilibrium and the rapid conversion of intermediates (*OOH) [26, 27], elevating the electrocatalytic activity. Thus, it is necessary to design a novel carbon‐based electrocatalyst with abundant active site and local rapid diffusion ability for efficient H_2_O_2_ production.

The controllable construction of carbon‐based nanoreactors (CNs) can achieve the effect of killing two birds with one stone, simultaneously adjusting the diffusion behavior of electrocatalysts at the mesoscale and offering a favorable electronic structure at the nanoscale [28]. Nanopore and geometry morphology engineering can effectively regulate the local mass transfer capacity of carbon materials [29, 30, 31]. Abundant reported studies have shown that large‐sized mesopores facilitate the exposure of the accessible active sites and the rapid removal of H_2_O_2_, avoiding excessive H_2_O_2_ concentrations in the electrode microenvironment [32, 33, 34, 35]. Meanwhile, a comprehensive understanding of the fluid behavior of CNs with different morphologies can also provide an important theoretical reference for the design of electrocatalysts. In addition, from a molecular design perspective, achieving the controllable synthesis of the bottom‐up in situ doped CNs by using heteroatom‐contained molecular as the precursor, which can change the spin or charge distribution of the sp^2^ carbon plane [36, 37], reduces the adsorption energy of oxygen‐containing species, thereby improving the intrinsic electrocatalytic selectivity. However, the lack of optional precursors and the difficulty in regulating the assembly kinetic and thermodynamic equilibrium between precursors and templates lead to challenges in the modulation of catalyst composition, the diversification of nano‐morphology, and pore structure. Moreover, the current researches on electrocatalytic diffusion behavior mainly focus on the macroscopic scale, but the theoretical development of rational manipulation process enhancement to improve catalytic performance at the mesoscale has not yet been fully discussed. These factors have severely hindered the study of the function and applicability of carbon materials in actual electrocatalytic synthesis. Therefore, a synthetic strategy that seamlessly integrates molecular‐level heteroatom doping to dictate the electronic structure with mesoscale geometry engineering to regulate mass transport represents a promising yet challenging path toward revolutionizing the electrocatalytic synthesis of H_2_O_2_.

Herein, an entropy‐driven interface self‐assembly strategy was proposed, by in situ polymerization of 3‐aminothiophenol (ATP) monomer at the oil‐water interface under mild conditions, a series of novel poly(3‐aminothiophenol) (PATP)‐based mesoporous polymeric particles (MPPs) and derived mesoporous carbon‐based particles (MCPs) with diverse morphologies and pore structures can be obtained. During this synthesis, the key point is to regulate the size of trimethylbenzene (TMB) oil droplets to accurately drive the self‐assembly process of the composite micelles at the oil‐water interface according to the system entropy stability effect, thus forming the lowest energy mesostructure. The morphology of the samples could be transformed from cavity nanospheres to mesoporous bowl‐like nanospheres to mesoporous gauze‐shaped nanosheets and mesoporous lotus leaf‐like nanosheets by altering the volume of TMB. The optimal sample achieves prominent intrinsic 2e^–^ ORR activity and selectivity in a rotating ring disk electrode (RRDE) test with an onset potential of 0.52 V versus reversible hydrogen electrode (RHE) and H_2_O_2_ selectivity of 91%. Impressively, this model catalyst exhibits a remarkable H_2_O_2_ yield of 15.8 mol_gcat_^−1^ h^−1^ at a current density of 200 mA cm^−2^ in the flow cell while maintaining high stability for 50 h. DFT calculations coupled with finite element analysis (FEA) simulations also reveal that the N,S dual heteroatoms can optimize the adsorption of *OOH, while the nanospheres with a bowl‐like opening structure facilitate mass transport and O_2_ enrichment.

## 2 Results and Discussion

**FIGURE 1:**
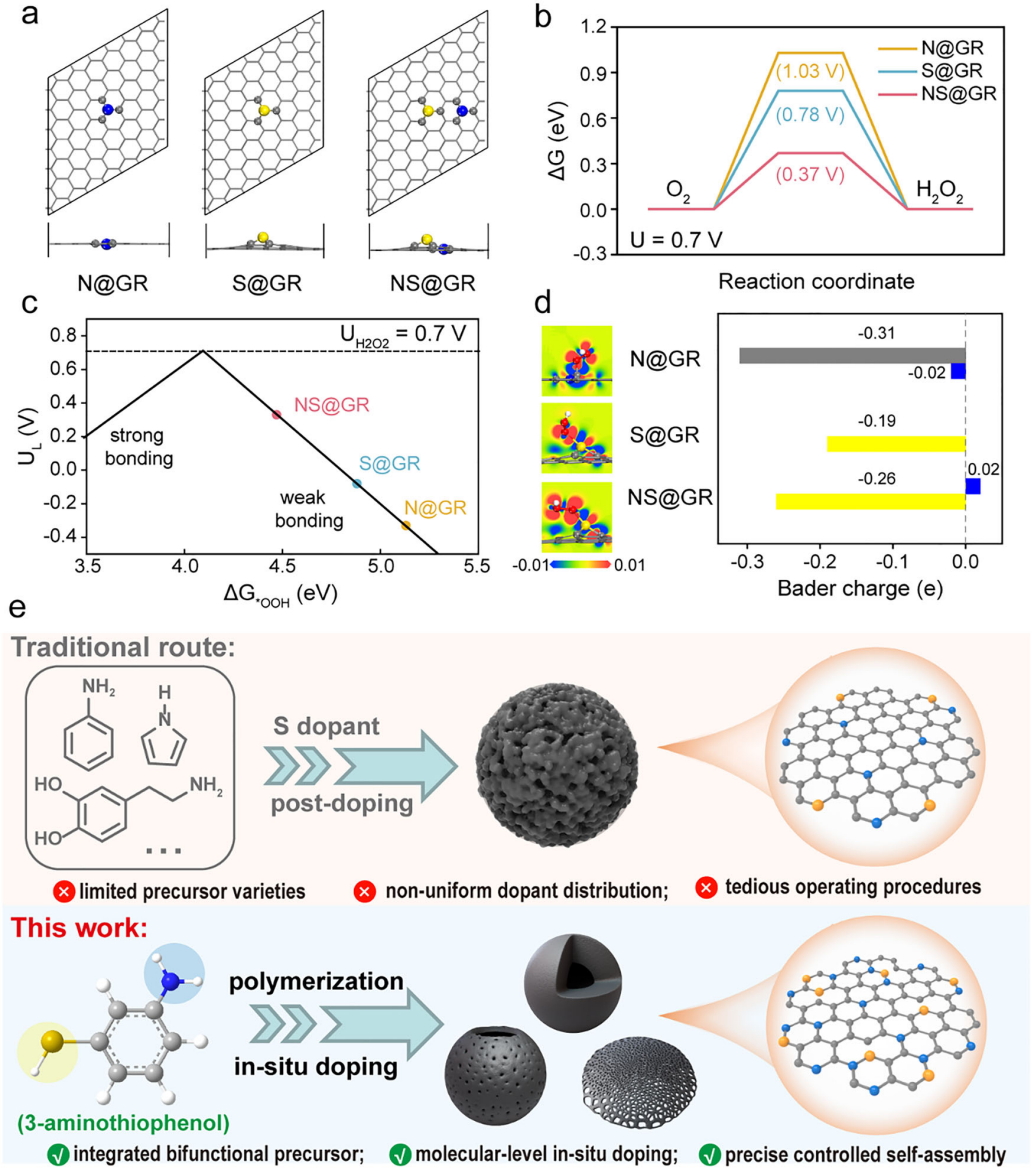
Theoretical predictions of catalytic performance and rational design and synthesis of catalysts. (a) Model structures of N@GR, S@GR and NS@GR. (b) Free energy diagrams of 2e^−^ ORR at 0.7 V. (c) Relationship between Δ*G* of the *OOH intermediate and the limiting potential (dots represent the positions of N@GR, S@GR, and NS@GR corresponding to the volcano plot). (d) Bader charge differences of the C/S/N atom (grey, yellow and blue column) in N@GR, S@GR, and NS@GR catalyst models adsorbed with and without *OOH, as well as the charge density differences of *OOH adsorbed on three models, in which the red and blue areas represent charge density accumulation and depletion, respectively. (e) Comparison between traditional post‐doping and proposed in‐situ molecular‐level doping for synthesizing mesoporous N,S‐dual‐doped carbons.

Dual heteroatom doping, particularly N,S‐dual‐doped carbon, has been widely used in the electrocatalytic production of H_2_O_2_. We first conducted density functional theory (DFT) calculations to predict the influence of heteroatoms on the 2e^−^ ORR performance of carbon. The different coordination structures of N atom, S atom, and N‐S atoms doped in the graphene (GR) are first constructed and compared (Figure S1), and the most stable catalyst models of N@GR, S@GR, and NS@GR are selected for the calculations in Figure 1a. The adsorption energy of the OOH intermediate is a key descriptor for the 2e^–^ ORR pathway of O_2_ + H_2_O → *OOH + OH^–^ → H_2_O_2_ + 2OH^–^. Thus, the Gibbs free energies of *OOH adsorption (ΔG_*OOH_) were investigated for the above three catalyst models at 0.7 V in Figure 1b (the calculation results at 0 V are shown in Figure S2), and a volcano plot relationship between the limiting potential (*U*_L_) and ΔG_*OOH_ is also described in Figure 1c. The overpotentials of 2e^–^ ORR for N@GR, S@GR, and NS@GR catalysts are 1.03, 0.78, and 0.37 V, respectively, indicating the N‐S di‐atomics dual‐doped catalyst exhibits superior electrocatalytic performance for the H_2_O_2_ formation compared to the singly doped catalysts. From the volcano plot, it can be observed that all three catalysts are located on the right side of the volcano, indicating there are weak interaction between *OOH and the three catalysts. In contrast, the NS@GR is much closer to the volcano apex (*U*_L_ = 0.7 V) than that of N@GR and S@GR, suggesting the N‐S co‐doping can obviously enhance the binding strength between *OOH and the catalyst. Furthermore, to elucidate the origin of the *OOH adsorption strength, the Bader charges of the different elements in the catalyst models of GR, N@GR, S@GR, and NS@GR, as well as the Bader charge differences (Δ*q*) for the active site of N@GR, S@GR, and NS@GR with and without *OOH adsorbed are analyzed. As the calculations show (Figure S3), the doped N atom (1.18 |e|) in N@GR can gain electrons from the C atoms (−0.31 |e|) bonded with it, while the electron can be transferred from the S atom (−0.45 |e|) to C atoms (0.23 |e|) in S@GR, suggesting the charge of GR can be redistributed both by the N and S atom, but they have the contrary electronic effect. Clearly, when the N (1.18 |e|) and S (−0.46 |e|) atom are dual‐doped in NS@GR, the charge is redistributed again and the electron density of C atoms becomes more unsymmetrical in the GR, including three states: –0.33 |e|, –0.02 |e|, and 0.22 |e|. For the top site adsorption of *OOH on the S atom in NS@GR and S@GR (Figure 1d, Table S1), it can be found that the Bader charge difference of the S atom bonded with and without *OOH in NS@GR (Δ*q* = –0.26 |e|) is more negative than that of S@GR (Δ*q* = –0.19 |e|), indicating the N atom in NS@GR can promote the electron transferring from the S atom to *OOH. For the N@GR, although the Bader charge difference of the C atom in N@GR (Δ*q* = –0.31 |e|) is more negative than that of S atom in NS@GR, the electron density of N atom in N@GR is reduced (Δ*q* = –0.02 |e|), which is not conducive to the stability of the N atom, because the N atom tends to gain electrons from GR. Fortunately, the Δ*q* of N atom in NS@GR is positive (0.02 |e|), indicating the electron density of N atom is not decreased but increased instead with the electron transferring from the S atom in NS@GR to *OOH, which leads to the more stable catalyst structure. Likewise, the charge density differences of *OOH adsorbed on N@GR, S@GR, and NS@GR are investigated as well, and the analysis has the same conclusion that the extent of the electron transfer from the catalysts to *OOH (the blue areas) follows the same order with the Bader charge analysis. In summary, the calculations demonstrate that, by modulating the electronic structure of the catalysts, the N/S co‐doping strategy can significantly enhance its electron‐donating capability toward the reaction intermediate (*OOH), and improve its 2e^−^ ORR performance.

Guided by theoretical predictions, we proposed an entropy‐driven interface self‐assembly strategy for preparing N,S‐dual‐doped MPPs and MCPs. Currently, most reported N,S‐dual‐doped carbon materials are synthesized via post‐doping, which involves the co‐pyrolysis of preformed nitrogen‐rich polymers (e.g., polyaniline, polypyrrole, polydopamine) with sulfur‐containing agents (e.g., S_8_, polyphenylene sulfide, thiourea) (Figure 1e). However, this traditional route suffers from limited precursor diversity, non‐uniform heteroatom doping, and tedious operating procedures. In this work, we strategically employ ATP as an integrated bifunctional precursor, containing both an amino group (‐NH_2_) and a sulfhydryl group (‐SH) within a single molecule. Using Pluronic F127 as structure‐directing agent and TMB as swelling agent, F127/TMB/ATP composite micelle can be formed through hydrogen bonding interaction between ‐NH_2_ and hydrophilic PEO segments [38, 39]. Subsequently, ammonium persulfate (APS) initiates the in situ chemical oxidation polymerization of ATP (Figure S4). By varying the amount of TMB, a series of MPPs are obtained (denoted as MPPs‐X, wherein X represents the volume of TMB), which are further pyrolyzed under a nitrogen atmosphere to yield the target MCPs. In contrast to the conventional method, this study not only achieves molecular‐level, in situ N,S‐dual‐doping in a single step but also enables precise control over the diverse assembly of the novel precursor.

**FIGURE 2:**
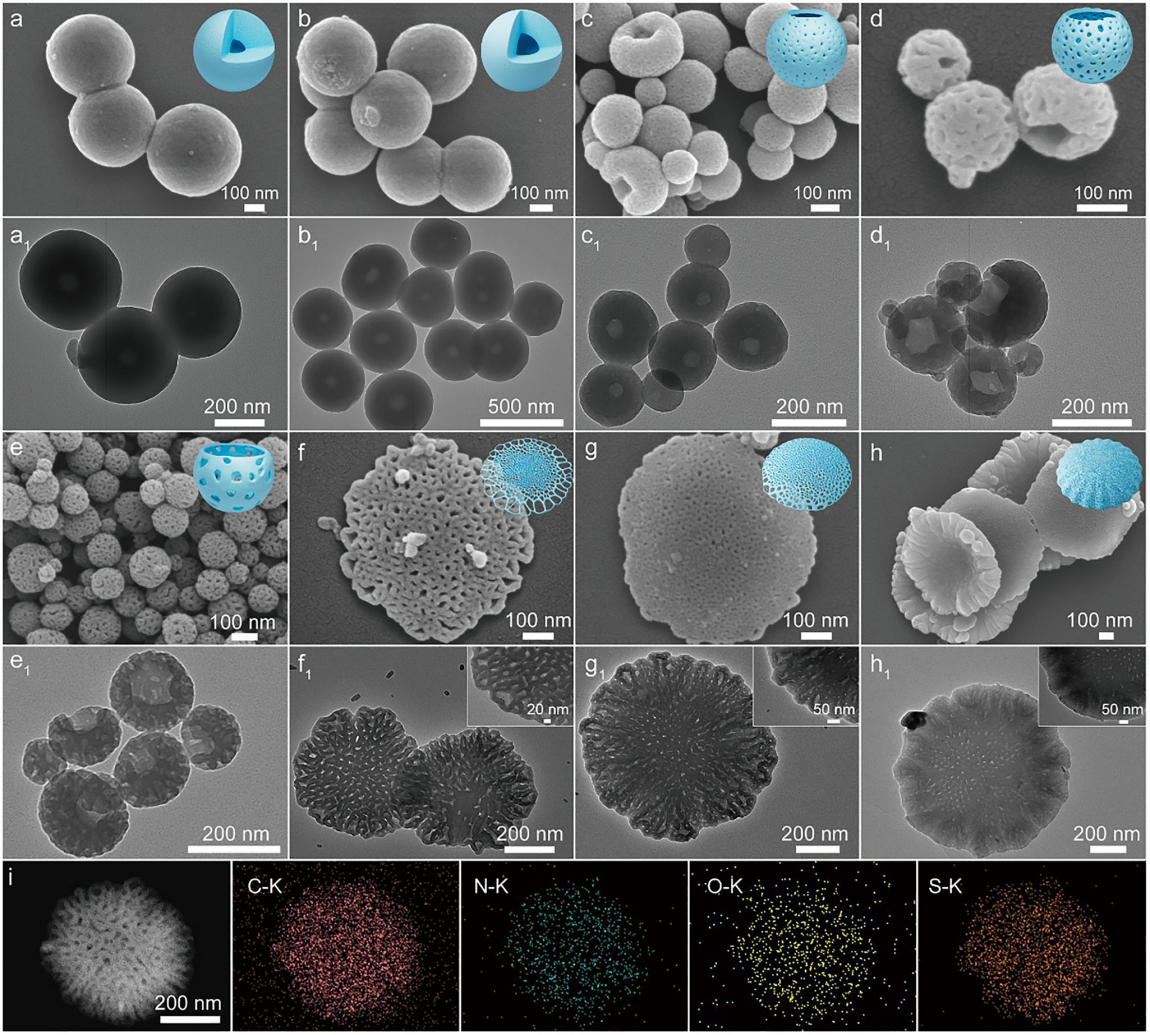
Characterization of morphological evolution for MPPs. (a–h) SEM images and (a_1_–h_1_) TEM images of MPPs by using different volume of TMB: (a, a_1_) MPPs‐0; (b, b_1_) MPPs‐0.1; (c, c_1_) MPPs‐0.2; (d, d_1_) MPPs‐0.3; (e, e_1_) MPPs‐0.4; (f, f_1_) MPPs‐0.8; (g, g_1_) MPPs‐1.5; (h, h_1_) MPPs‐2.0. (i) EDX elemental maps of MPPs‐0.8.

The morphology and structure of MPPs were further characterized by scanning electron microscope (SEM) and transmission electron microscopy (TEM). It is worth mentioning that the appropriate content of TMB plays an indispensable role in the formation of diversified MPPs. When the system lacked TMB, SEM image of MPPs‐0 exhibited a well‐defined nanosphere morphology with rough surface and an average diameter of 420 nm (Figures 2a and S5). Interestingly, TEM image showed a small cavity of about 40 nm in the center of the nanosphere (Figure 2a_1_). Compared with MPPs‐0, the size of nanosphere MPPs‐0.1 was slightly reduced, but the cavity size was significantly increased (Figures 2b,b1 and S6). Next, as the TMB volume increased, the morphology of the samples evolved from cavity nanospheres to small‐sized mesoporous bowl‐like nanospheres (about 164– 221 nm). TEM images revealed that the size of the bowl mouth gradually enlarged while the mesopores on the bowl walls became more pronounced (Figures 2c–e,c1–e1 and S7), which suggested that the swelling effect on F127 micelles progressively intensified with increasing TMB dosage. Intriguingly, when the TMB volume reached 0.8 and 1.5 mL, two structurally distinct mesoporous gauze‐like nanosheets with diameters of approximately 500 ∼ 800 nm were obtained (Figure 2f,g,f1,g1 and S8). SEM and TEM images clearly demonstrated the presence of a mesopore structure, with the nanosheet edges displaying curled and wrinkled configurations. Notably, MPPs‐1.5 exhibited a significantly higher porosity compared to MPPs‐0.8. Furthermore, mesoporous lotus‐leaf‐like nanosheets were successfully fabricated upon introducing 2.0 mL of TMB into the system. Remarkably, structural characterization revealed an abundance of mesopores on the convex side of the nanosheets, whereas the concave side remained devoid of apparent pore structures, accompanied by obvious wrinkling along the edges (Figures 2h,h1 and S9). Ultimately, even with excessive TMB dosage (3.0 mL), the sample maintained its lotus‐leaf‐like morphology (Figure S10). Moreover, several control experiments were systematically conducted following the MPPs synthesis method. In the absence of both F127 and TMB, ATP monomers underwent spontaneous polymerization, yielding irregular and severely viscous particles. When solely introducing TMB (0.8 mL), the sample appeared as adhesive sheets and particles (Figure S11). In conclusion, our findings highlight that a well‐balanced F127/TMB composition plays a pivotal role in governing the evolution of morphology and pore architecture in MPPs, where their synergistic interaction dictates structural hierarchy across multiple length scales. As shown in the corresponding element mapping images (Figures 2i and S12), C, N, O, and S elements were evenly distributed in the whole polymeric frameworks.

As widely acknowledged, ethanol/water mixture serves not merely as the aqueous phase for emulsification, but also functions as crucial co‐solvent for regulating the morphology of nanoparticles [40, 41, 42]. Here, we initially investigated the effect of ethanol volume fraction on the morphology evolution of nanoparticles under TMB‐free conditions. Intriguingly, as the volume fraction diminished from 65% to 35%, the prepared polymeric nanospheres exhibited precisely tunable dimensions spanning 153 to 678 nm, which may be related to the effect of ethanol content on monomer polymerization rate (Figure S13). Additionally, a series of alternative experiments were carried out based on the synthesis process of MPPs. Initially, by substituting the structure‐directing agent F127 with equivalent amounts of F108 or F68, mesoporous nanosheets architecture that closely resembled MPPs‐0.8 and folded nanosheets were obtained, respectively (Figure S14). Second, replacing TMB as the primary oil phase with n‐hexane and n‐hexanol resulted in smooth lotus‐leaf‐like nanosheet structures that akin to MPPs‐2.0 and uniform nanospheres (Figure S15). These results may originate from the distinct hydrophobic block lengths in various structure‐directing agents and the swelling capacity of different oil phases.

**FIGURE 3:**
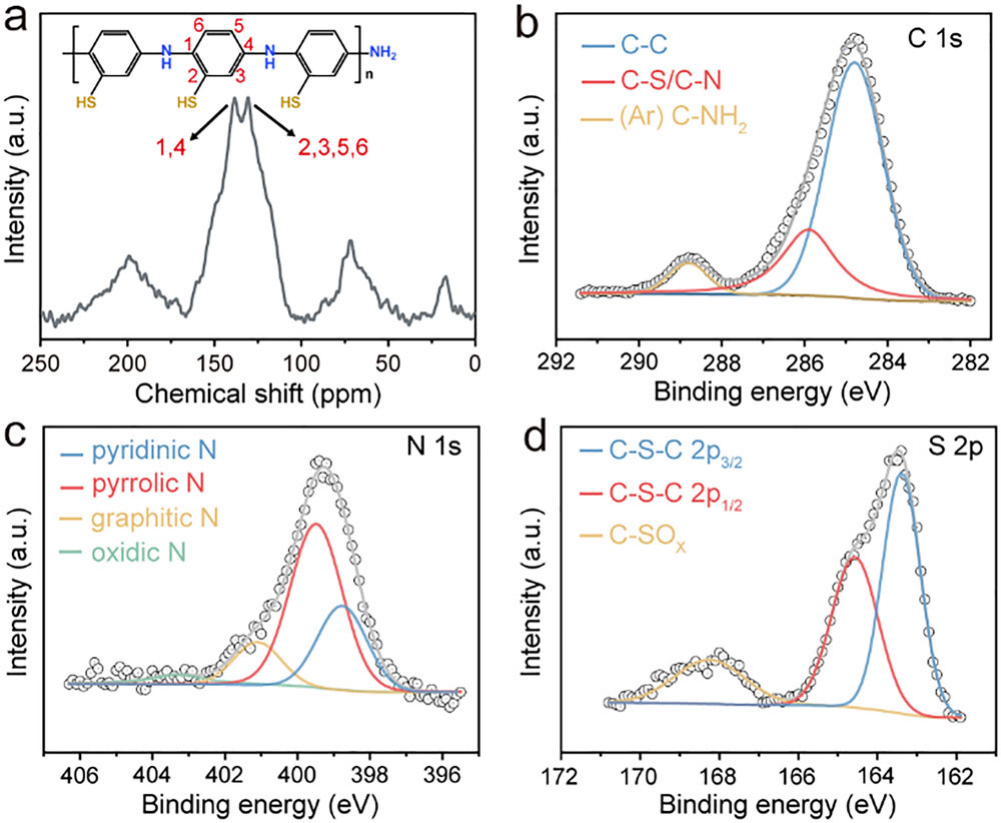
Characterization of physical and chemical properties of MPPs. (a) ^13^C (CP‐MAS) NMR spectrum, (b–d) high‐resolution XPS spectra of C 1s, N 1s, and S 2p, respectively.

The chemical structure of the as‐prepared MMPs can be determined by combining the Fourier‐transform infrared (FT‐IR) spectrum with ^13^C cross‐polarization magic‐angle spinning (CP/MAS) NMR spectrum. As depicted in Figure S16, a series of distinguishable absorption peaks corresponding to chemical bond characteristics within the polymeric framework were clearly identified. Typically, the absorption peaks at 1280 and 2760 cm^−1^ belong to the stretching vibration of C‐N and S‐H, respectively. The absorption band at 3350 cm^−1^ is classified as the stretching vibration of N–H, indicating the presence of secondary amine structures and the successful transformation of ATP monomers into a PATP‐based framework. In addition, no significant characteristic adsorption band of C‐H at 2884 cm^−1^ in F127 was detected in the polymer skeleton [43, 44], confirming the successful removal of the templating agent. The ^13^C (CP‐MAS) NMR spectrum in Figure 3a shows two obvious resonances related to polymer components at 130 and 139 ppm, which are assigned to carbon atoms in distinct chemical environments within the aromatic ring. To further delve the chemical structure information on the as‐prepared MPPs, the x‐ray photoelectron spectroscopy (XPS) was employed. The XPS survey spectrum exhibits four obvious peaks around 285, 399, 532.5, and 164 eV, which are ascribed to C 1s, N 1s, O 1s, and S 2p, respectively (Figure S17). It is further demonstrated that the corresponding surface atomic percentage is 50.1% (C), 5.0% (N), 39.0% (O), and 5.9% (S), respectively. The high‐resolution C 1s spectrum can be deconvoluted into three peaks corresponding to functional groups C‐C (284.8 eV), C‐S/C‐N (285.9 eV), and (Ar) C‐NH_2_ (288.8 eV) [45, 46], respectively (Figure 3b). Furthermore, the N configuration can be mainly divided into four peaks including pyridinic N (398.6 eV), pyrrolic N (399.5 eV), graphitic N (401.2 eV) and oxidic N (403.3 eV) (Figure 3c) [47, 48]. As performed in Figure 3d, the high‐resolution S 2p spectra shows three typical peaks, where the peaks at 163.4 and 164.6 eV can be assigned to S 2p_3/2_ and S 2p_1/2_ of C–S bond, and the peak at 168.3 eV can be attributed to C‐SO_x_ [49, 50]. The above results reveal that the polymer materials maintain considerable heteroatom doping characteristics, suggesting N and S elements are successfully in‐situ doped in the polymer skeleton.

As performed in Figure S18, the XRD pattern manifests that there are two broad diffraction peaks containing typical (002) and (100) diffraction planes, suggesting the amorphous structure of MPPs. In addition, the porosity of material was further investigated through nitrogen adsorption technology. The nitrogen adsorption‐desorption isotherms of MPPs show an obvious hysteresis loop in the middle‐high pressure range (*P*/*P*_0_ = 0.4–0.8) and can be classified as the typical type‐ IV curves [51], indicating the presence of mesopores (Figure S19). The Brunauer–Emmett–Teller (BET) specific surface area of MPPs range from 12–91 m^2^ g^−1^. The pore size distribution based on Barrett–Joyner–Halenda (BJH) model suggests the presence of micropores and mesopores, with the mesopores mainly concentrated in the range of 2.2–12.3 nm, which is consistent with TEM observations. All the related structural parameters of different samples are summarized in Table S2.

**FIGURE 4:**
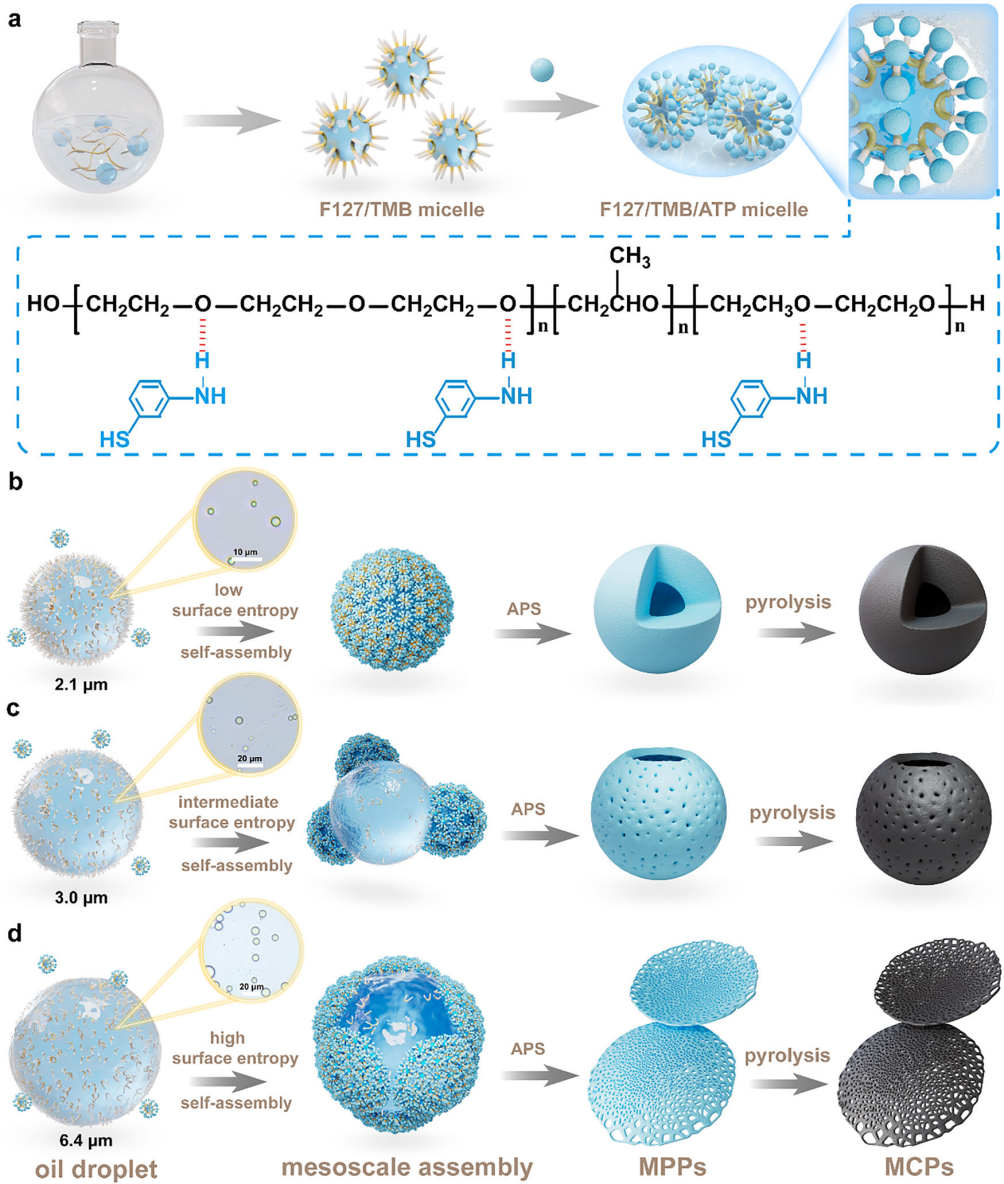
Proposed formation mechanisms of MPPs and MCPs via entropy‐driven interface self‐assembly strategy.

On the basis of the results and analyses aforementioned, an entropy‐driven interface self‐assembly strategy is proposed for preparing MPPs and MCPs. In the synthetic system, a well‐defined oil‐in‐water biphasic emulsion system was initially formed, comprising hydrophobic TMB molecules, amphiphilic F127 template, and ATP monomers. It is well known that the F127 copolymer can self‐assemble into spherical micelles with a hydrophobic PPO core and a hydrophilic PEO corona in an aqueous solution. The hydrophobic TMB molecules exhibit a favorable swelling effect as the “like dissolves like” principle, preferentially interacting with the hydrophobic PPO blocks through van der Waals force, thereby expanding the spherical micelles to form F127/TMB micelles (Figure 4a). Further, ATP monomers are sequentially arranged at the oil–water interface through hydrogen bonding interaction between ‐NH_2_ and hydrophilic PEO segments, forming F127/TMB/ATP composite micelles (denoted as component A; Figure S20). Additionally, the mixture of TMB with ethanol/water can also directly form F127‐stabilized TMB oil droplets (denoted as component B) to create oil/water interfaces for micelle anisotropic self‐assembly. Specifically, ATP monomers exhibit inherent hydrophobicity within the system (Figure S21). Driven by strong van der Waals and lipophilic interaction, component A tends to be assembled on component B. When the TMB volume is 0–0.1 mL (Figure 4b), component B exhibits a relatively small size (2.1 µm) that can only provide a limited assembly interface. This configuration represents a state of relatively low surface entropy. To maintain the entropy stability effect of the system, component A will continuously deposit and assemble on the surface of component B during this stage and undergo gradual transformation into a mesostructured micelle array through full‐coverage and close‐packing way, forming the lowest energy state. Subsequently, in the presence of oxidant APS, ATP monomers undergo in situ chemical oxidative polymerization to form a PATP‐based polymeric skeleton. Following the removal of component B, mesoporous polymeric nanospheres with cavities are successfully obtained. As the TMB volume increases to 0.2–0.4 mL (Figure 4c), component B exhibits a significant volumetric expansion (3.0 µm) (Figure S22), generating a greater number of assembly interfaces. This morphology exhibits an intermediate surface entropy, and component A tends to aggregate and selectively embed partially onto the surface of component B in the form of multiple discrete micelle nano‐islands for subsequent polymerization. Upon removal of TMB, mesoporous bowl‐shaped nanospheres are obtained. The swelling effect is enhanced with increasing TMB volume, resulting in progressive enlargement of the mesoporous size of the bowl mouth and the bowl wall. When the TMB volume increases to 0.8–2.0 mL (Figure 4d), component B attains larger mean size (6.4 µm) compared to the two aforementioned scenarios. Under such circumstances, component A assembles by forming multiple nanosheet arrays that cover the surface of component B. While the formation of large, 2D sheets might seem like an ordered, low‐entropy state, it is actually a result of an entropy‐driven escape from an unfavorable high‐energy interface. Concurrently, owing to the expanded assembly interface, more component A will accumulate at the oil–water interface, accompanied by progressive enhancements in both the packing density and thickness of the nanosheets. Once these sheets form and eventually detach from the oil droplet, the interconnected nanosheet architectures on the surface of component B are exfoliated into individual nanosheets including mesoporous gauze‐like nanosheets and mesoporous lotus‐leaf‐like nanosheets. Finally, the obtained MPPs were calcined and further converted into nitrogen‐sulfur dual‐doped MCPs. To sum up, it is unquestionably ingenious to precisely regulate the diverse assembly of composite micelles through entropy‐driven strategies, which offers a straightforward pathway for synthesizing unique anisotropic nanoparticles.

PATP serves as a novel nitrogen and sulfur dual‐doped precursor. Subsequently, three representative MPPs (MPPs‐0.2, MPPs‐0.8, and MPPs‐1.5) were selected and carbonized at 600°C under N_2_ atmosphere to produce MCPs‐X, wherein X represented the volume of TMB. TEM images revealed that MCPs successfully retained the morphology and pore structures of MPPs (Figure S23). Thermogravimetric analysis (TGA) indicated that the carbonization yields of PATP at 600°C was approximately 51 wt% (Figure S24). The N_2_ adsorption–desorption isotherms of the MCPs (Figure S25) showed strong uptakes at low pressures (*P*/*P*_0_ < 0.1), demonstrating the presence of micropores in all the samples, which most probably originated from the polymer degradation. Meanwhile, MCPs exhibited a typical type‐IV adsorption–desorption isotherm with a hysteresis loop, suggesting the presence of mesopores. The BET specific surface area of MCPs‐0.2, MCPs‐0.8, and MCPs‐1.5 were calculated to be 519.7, 556.2, and 569.2 m^2^ g^−1^, respectively (Table S3). Moreover, XPS analysis revealed characteristic peaks corresponding to C 1s, N 1s, O 1s, and S 2p in the MCPs (Figure S26), with their elemental contents being relatively close. Additionally, XPS characterization was also performed on the samples calcined at 700°C and 800°C. The results showed a continuous decreasing trend in the contents of N and S within the carbon framework as the pyrolysis temperature elevated. Notably, the S content decreased from 1.69% to 1.31%, while the N content also decreased to 2.14% at 800°C, suggesting that the N and S active sites in the carbon framework are susceptible to loss at increased pyrolysis temperatures (Figure S27 and Table S4).

**FIGURE 5:**
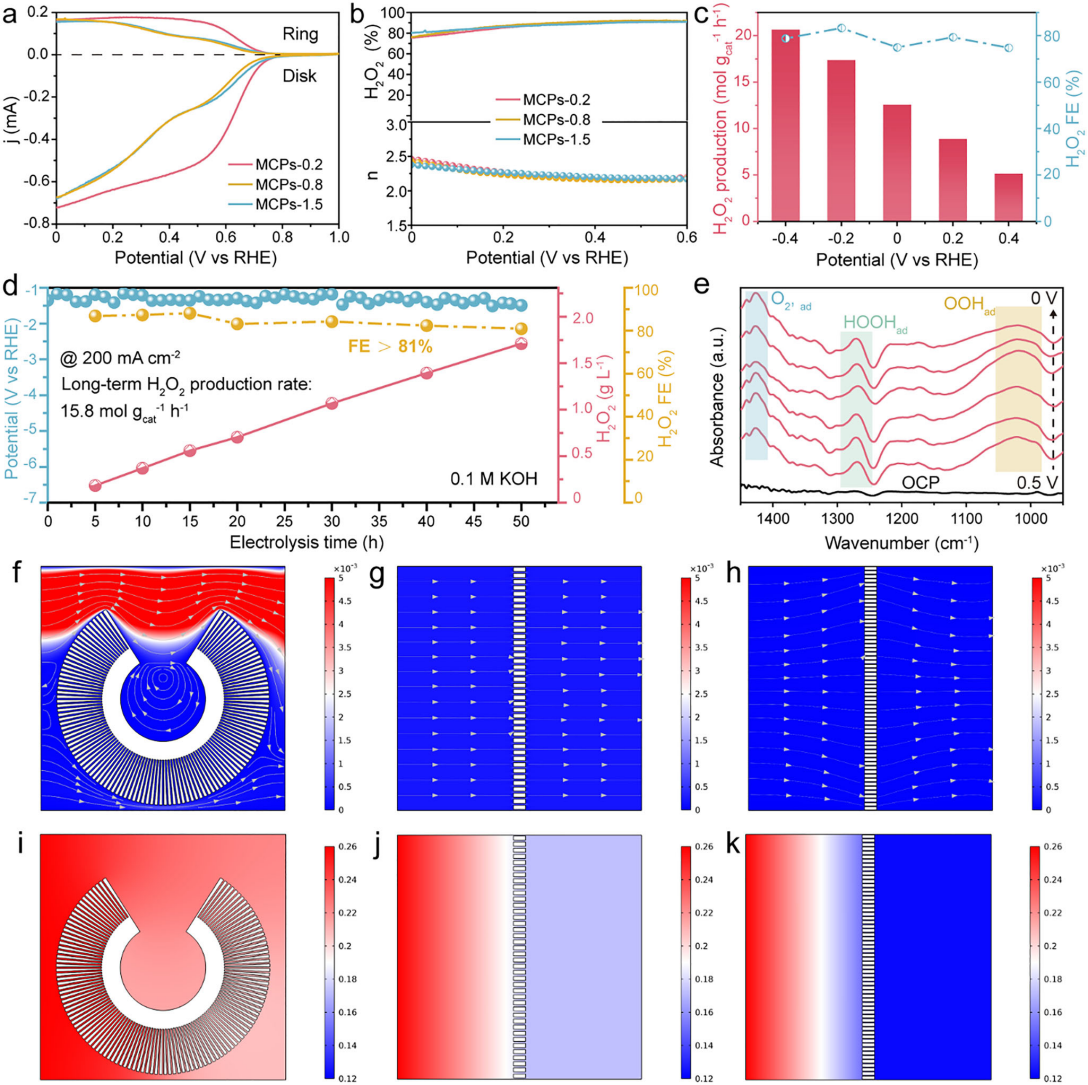
Electrochemical H_2_O_2_ production performance and FEA simulations. (a) LSV curves recorded on the RRDE in O_2_‐saturated 0.1 M KOH at 1600 rpm. (b) H_2_O_2_ selectivity and electron transfer number. (c) H_2_O_2_ production rates and FEs of MCPs‐0.2 at different potentials. (d) Electrolysis time‐dependent H_2_O_2_ production rates, FEs and accumulated H_2_O_2_ concentration on MCPs‐0.2 at 200 mA cm^−2^ under continuous O_2_ purging in flow cell. (e) In situ ATR‐SEIRAS spectra for MCPs‐0.2 in O_2_‐saturated 0.1 M KOH. (f–h) Simulated spatial distribution of flow velocities over different models. (i–k) Simulated spatial distribution of O_2_ concentration over different models. Colors represent corresponding numerical values.

As a proof of concept, these novel MCPs were applied as metal‐free electrocatalysts for the electrochemical 2e^−^ ORR. The intrinsic electrochemical performance was evaluated using a three‐electrode RRDE configuration in an O_2_‐saturated 0.1 M KOH electrolyte. Here, the samples pyrolyzed at 600°C were selected for subsequent testing. Figure 5a exhibits ORR polarization curves recorded by linear sweep voltammetry (LSV) at the RRDE rotation speed of 1600 rpm. All samples show the obviously high ring current with an equivalent disk current, highlighting their tendency toward a two‐electron pathway. Besides, the MCPs‐0.2 shows high H_2_O_2_ electrosynthesis activity as evidenced by their onset potential of 0.78 versus RHE, which is close to the thermodynamic equilibrium potential for 2e^−^ ORR [52, 53, 54]. As shown in Figure 5b, all MCPs samples exhibit impressive H_2_O_2_ selectivity (over 90%) within a wide potential window (0.3–0.6 V vs. RHE), with an electron transfer number (*n*) close to the ideal value of 2. Their selectivity values are superior to those of the samples calcined at 700°C and 800°C (Figure S28), indicating that the proportion of N and S active sites within the carbon framework plays a crucial role in regulating the ORR pathway. Furthermore, compared to MCPs‐0.8 (122.9 mV dec^−1^) and MCPs‐1.5 (117.8 mV dec^−1^), MCPs‐0.2 exhibits the lowest Tafel slope (83.7 mV dec^−1^) (Figure S29). This confirms its rapid 2e^−^ ORR reaction kinetics, which may originate from its appropriate porous structure and geometric framework that enhance contact with the electrolyte fluid, thereby improving the utilization of active sites on the carbon surface in the electrolyte environment. Inspired by the remarkable 2e^−^ ORR selectivity and H_2_O_2_ production capacities of the prepared catalysts, we further evaluated the corresponding electrocatalytic efficiency for H_2_O_2_ generation in a flow‐cell mode. In this configuration, we initially performed electrolysis experiments at various applied potentials (−0.4 to 0.4 V vs. RHE) and current densities (25–200 mA cm^−2^) to assess the H_2_O_2_ production. As shown in Figures 5c and S30, the H_2_O_2_ yield increases linearly with a negative shift in voltage, while the Faradaic efficiency (FE) exhibits only a slight decrease. At −0.2 V versus RHE, the catalyst MCPs‐0.2 achieves an H_2_O_2_ production rate of up to 17.38 mol_gcat_^−1^ h^−1^, with an FE still as high as 83.3%, which is significantly better than that of MCPs‐0.8 (13.97 mol_gcat_^−1^ h^−1^, 75.1%) and MCPs‐1.5 (9.06 mol_gcat_^−1^ h^−1^, 42.0%). Meanwhile, the FE of H_2_O_2_ remains at approximately 80% across the applied current density range, and the yield of H_2_O_2_ reaches 16.13 mol_gcat_^−1^ h^−1^ at 200 mA cm^−2^ (Figure S31). To investigate the stability of MCPs‐0.2, a 50‐h continuous electrolysis was conducted at 200 mA cm^−2^ in a 0.1 M KOH solution (Figure 5d). During this process, MCPs‐0.2 exhibits a stable voltage with a sustained H_2_O_2_ production rate of 15.8 mol_gcat_^−1^ h^−1^ and an overall FE exceeding 81%. And the cycled catalyst samples remain stable in terms of morphology, pore structure, and composition (Figure S32), demonstrating that the catalyst possesses excellent durability and stability. Furthermore, to study the relationship between selectivity and the adsorbed species on the catalyst surface at different reaction potential, in situ attenuated total reflectance surface‐enhanced infrared absorption spectroscopy (ATR‐SEIRAS) was carried out on MCPs‐0.2. The in situ ATR‐SEIRAS spectrum were recorded by varying the potential stepwise from 0.5 to 0 V versus RHE in O_2_‐saturated 0.1 M KOH. As displayed in Figure 5e, three absorption peaks related to oxygen‐containing species adsorbed on MCPs‐0.2 can be clearly observed. Specifically, the band observed at about 1023 cm^−1^ is attributed to the O‐O stretching vibration of surface‐adsorbed superoxide (OOH_ad_). The band at 1272 cm^−1^ arises from the OOH bending mode of adsorbed hydroperoxide (HOOH_ad_), while the feature at 1426 cm^−1^ could be assigned to the O‐O stretch of weakly adsorbed molecular oxygen (O_2_, _ad_) [55, 56, 57, 58]. These results indicate that N doping is primarily responsible for enhancing the overall ORR activity and onset potential, while S doping helps to suppress the unfavorable 4e^−^ pathway and promote the selective generation of H_2_O_2_. The synergistic effect of the two enables the dual‐doped system to achieve an ideal balance in performance. Meanwhile, under the same testing conditions, we evaluated the performance of a series of other control samples, including single‐doped N‐doped carbon, S‐doped carbon, and post‐treated N,S‐dual‐doped carbon. Compared with MCPs‐0.2, these control samples exhibit lower selectivity and activity, along with weaker signals of relevant intermediates detected by in situ ATR‐SEIRAS (Figure S33), indicating that the molecular‐level in situ N,S‐dual‐doped carbon material is more advantageous for the electrosynthesis of H_2_O_2_. In the end, to further illustrate the outstanding performance of material, the result was compared with the recently reported 2e^–^ ORR electrocatalysts including nonprecious metal, and metal‐free carbon‐based catalysts (Table S5), MCPs‐0.2 exhibits a high H_2_O_2_ selectivity under the alkaline solution. In addition to investigating the N,S‐dual‐doping effect at the atomic scale, we also attempted to reveal the influence of the mesoscale morphological structure. First, electrochemical impedance spectroscopy (EIS) analysis was performed (Figure S34). The EIS spectra showed that the MCPs‐0.2 electrode exhibited lower impedance than the MCPs‐0.8 and MCPs‐1.5 electrodes, which may be attributed to its unique bowl‐shaped structure and suitable mesoporous structure. Furthermore, FEA was employed for simulation. The simulation results indicate that mesoporous carbon nanospheres with a bowl‑like open structure exhibit significantly higher fluid velocity on their surface (Figure 5f), while nanosheet structures with different pore sizes demonstrate lower surface flow velocities (Figure 5g,h). This velocity gradient directly affects the mass transfer efficiency of reactants. Quantitative analysis of the flow distribution further confirms that MCPs‑0.2 possesses the optimal mass transfer characteristics (Figure S35). Hydrodynamic simulations reveal that the enhanced mass transfer process also effectively promotes the adsorption of O_2_ on the catalyst surface, thereby maximizing the overall reaction activity of 2e^−^ ORR (Figures 5i–k and S36).

## 3 Conclusion

In summary, DFT calculations predict that N,S‐dual‑doping optimizes *OOH adsorption in H_2_O_2_ electrosynthesis. Guided by this insight, an entropy‑driven interfacial self‑assembly strategy was developed to synthesize carbon materials with in situ molecular‑level N,S‐dual‑doping. Furthermore, varying the TMB dosage allowed for precise and versatile control over the nanomorphology and mesoporous structure of the MCPs. As a proof of concept, the optimal catalyst MCPs‐0.2 exhibited high H_2_O_2_ selectivity (91%) in alkaline electrolytes, and showed the yield of up to 17.38 mol_gcat_^−1^ h^−1^ at −0.2 V versus RHE potential and up to 16.13 mol_gcat_^−1^ h^−1^ at 200 mA cm^−2^ in a flow cell device, suggesting its superior application potential in H_2_O_2_ production. FEA simulations further indicate that specific mesostructures can effectively enhance mass transport and O_2_ enrichment, thereby improving reaction kinetics. This work not only provides fundamental insights into the structure‐performance relationship of metal‐free electrocatalysts at both atomic and mesoscale levels but also offers a novel guideline for the development of application‐oriented H_2_O_2_ electrocatalysts.

## Author Contributions

**Fei Liu**: writing – original draft, formal analysis, investigation, methodology. **Xiaoqing Liu**: formal analysis, validation. **Rui Zhang**: writing – review and editing, investigation, funding acquisition, supervision. **Linxia Cui**: software. **Rui Gao**: software, writing – review and editing. **Ji Liang**: resources. **Jian Liu**: funding acquisition, writing – review and editing, supervision.

## Conflicts of Interest

The authors declare no conflicts of interest.

## Supporting information




**Supporting File 1**: Anie72857‐sup‐0001‐SuppMat.docx.

## Data Availability

The data that support the findings of this study are available in the Supporting Information of this article.
