# Supplementary material for: Mesoporous N,S‐Dual‐Doped Carbon Nanoreactors via Entropy‐Driven Interface Self‐Assembly for Efficient H2O2 Electrosynthesis
Source: Angew Chem Int Ed Engl. 2026 May 23;65(30):e7636911. doi: 10.1002/anie.7636911 (PMC13383176; doi:10.1002/anie.7636911)
Supplement: Supplementary file 1 — Supporting File 1: Anie72857‐sup‐0001‐SuppMat.docx. [file ANIE-65-e7636911-s001.docx]

**Supporting Information – Table of Contents**

**Experiment Section**

Chemicals and Materials

Synthesis of the mesoporous polymeric particles (MPPs)

Synthesis of the mesoporous carbon-based particles (MCPs)

Synthesis of control samples

N-doped carbon

S-doped carbon

N,S-co-doped carbon

Characterizations

Electrochemical Measurements

Computational details

Finite element analysis simulations

**Figures**

**Figure S1**: Atomic models of N‑doped, S‑doped, and N,S‑dual‑doped graphene with binding energies

**Figure S2**: Gibbs free energy diagrams of 2e⁻ ORR pathways

**Figure S3**: Bader charge analysis of pristine and doped graphene

**Figure S4**: Schematic of chemical oxidative polymerization mechanism of ATP

**Figure S5**: External/internal diameter of MPPs‑0

**Figure S6**: External/internal diameter of MPPs‑0.1

**Figure S7**: TEM images of MPPs‑0.2, MPPs‑0.3, MPPs‑0.4

**Figure S8**: TEM images of MPPs‑0.8 and MPPs‑1.5

**Figure S9**: SEM/TEM images of MPPs‑2.0

**Figure S10**: SEM/TEM images of MPPs‑3.0

**Figure S11**: SEM/TEM of samples without F127/TMB or with only TMB

**Figure S12**: EDS element distribution

**Figure S13**: SEM/TEM with varied ethanol content

**Figure S14**: SEM/TEM of samples using F108 and F68 as structure-directing agents

**Figure S15**: SEM/TEM of samples using n‑hexane and n‑hexanol as oil phase

**Figure S16**: FT‑IR spectra of PATP, F127/PATP and F127

**Figure S17**: XPS survey spectrum and element mass percentages

**Figure S18**: XRD pattern

**Figure S19**: Nitrogen adsorption‑desorption isotherms and pore size distribution

**Figure S20**: Optical photographs of F127/ATP/TMB system (Tyndall effect)

**Figure S21**: Optical photographs of ATP monomer in different phases

**Figure S22**: Optical micrographs of F127‑stabilized TMB oil droplets

**Figure S23**: TEM images of MCPs‑0.2, MCPs‑0.8, MCPs‑1.5

**Figure S24**: TGA curves of PATP and F127

**Figure S25**: N_2_ adsorption‑desorption isotherms of MCPs‑0.2, MCPs‑0.8, MCPs‑1.5

**Figure S26**: XPS spectra of MCPs‑0.2, MCPs‑0.8, MCPs‑1.5 (600 °C)

**Figure S27**: XPS spectra of MPPs‑0.2 pyrolyzed at 700 °C and 800 °C

**Figure S28**: H_2_O_2_ selectivity vs. pyrolysis temperature

**Figure S29**: Tafel plots from LSV curves

**Figure S30**: H_2_O_2_ production rates and FEs at different potentials (MCPs‑0.8, MCPs‑1.5)

**Figure S31**: H_2_O_2_ production rates and FEs at different current densities

**Figure S32**: Post‑stability characterization of MCPs‑0.2

**Figure S33**: Control samples (N‑doped, S‑doped, N,S‑co‑doped): TEM, LSV, selectivity, production rates, in situ ATR‑SEIRAS

**Figure S34**: EIS plots of MCPs‑0.2, MCPs‑0.8, MCPs‑1.5

**Figure S35**: Simulation results-flow rate

**Figure S36**: Simulation results-O_2_ concentration distribution

**Tables**

**Table S1**: Bader charges of N@GR, S@GR, NS@GR models with/without *OOH adsorption

**Table S2**: Structural parameters of MPPs

**Table S3**: Structural parameters of MCPs at 600 °C

**Table S4**: Elemental content at different pyrolysis temperatures

**Table S5**: Comparison of 2e⁻ ORR performance with recently reported electrocatalyst

**Supporting Information**

**Experiment Section**

**Chemicals and Materials**

Triblock copolymer poly(ethylene oxide)-b-poly(propylene oxide)-b-poly(ethylene oxide) (Pluronic F127, PEO_106_-PPO_70_-PEO_106_), poly(ethylene oxide)-b-poly(propylene oxide)-b-poly(ethylene oxide) (Pluronic F108, PEO_132_-PPO_50_-PEO_132_), poly(ethylene oxide)-b-poly(propylene oxide)-b-poly(ethylene oxide) (Pluronic F68, PEO_80_-PPO_30_-PEO_80_), 1,3,5-trimethylbenzene (TMB), 3-aminothiophenol (C_6_H_7_NS), potassium hydroxide (KOH) and potassium titanium oxalate (K_2_TiO(C_2_O_4_)_2_) were purchased from Aladdin-Reagent Co. Ltd. Ammonium persulfate ((NH_4_)_2_S_2_O_8_, APS), hydrochloric acid (HCl, 37 %), isopropanol (C_3_H_8_O), ethanol (C_2_H_6_O) and sulfuric acid (H_2_SO_4_) were purchased from Fengchuan-Reagent Co. Ltd (Tianjin). Nafion (5 wt%) was purchased from Sigma-Aldrich. Deionized water was used for all experiments. All reagents were of AR grade quality and used without further purification unless otherwise mentioned.

**Synthesis of the mesoporous polymeric particles (MPPs)**

In a typical synthesis, firstly, Pluronic F127 (0.063 mmol) was dispersed in deionized water (20 mL) and ethanol (20 mL) and stirred until completely dissolved at room temperature. Then, a certain amount 3-aminothiophenol, TMB and 1 M HCl solution were added into the above mix solution and stirred for 1 h. Finally, appropriate amount of APS aqueous solution (130 mg/mL) was slowly added and stirred to polymerize for 24 h. The suspension was separated by centrifugation, washed with water and ethanol for three times. Finally, the product MPPs was dried in an oven at 60°C for 24 h.

**Synthesis of the mesoporous carbon-based particles (MCPs)**

Furthermore, a certain amount of dried MPPs was placed in a tube furnace, and then heated at a rate of 2°C/min in a nitrogen atmosphere to the appropriate temperature. The process was continued for 2 h to obtain MCPs.

**Synthesis of control samples**

**For N-doped carbon**: 0.4 g of F127 and 0.2 g of aminophenol were dissolved in 20/20 mL of ethanol and water under stirring. After complete dissolution, 1 mL of ammonia solution was added. After thorough stirring, 0.28 mL of formaldehyde was added. The mixture was stirred for 24 h, then washed with ethanol/water, and dried in an oven at 40 °C for 24 h. Subsequently, the obtained product was pyrolyzed under nitrogen atmosphere at 600 °C for 2 h with a heating rate of 2 °C/min.

**For S-doped carbon**: 0.4 g of F127 and 0.2 g of resorcinol were dissolved in 20/20 mL of ethanol and water under stirring. After complete dissolution, 1 mL of ammonia solution was added. After thorough stirring, 0.28 mL of formaldehyde was added. The mixture was stirred for 24 h, then washed with ethanol/water, and dried in an oven at 40 °C for 24 h. A small amount of sulfur powder (mass ratio of polymer to sulfur = 1:10) was added and thoroughly ground. The resulting mixture was then pyrolyzed under nitrogen atmosphere at 600 °C for 2 h with a heating rate of 2 °C/min.

**For N,S-co-doped carbon**: 0.4 g of F127 and 0.2 g of aminophenol were dissolved in 20/20 mL of ethanol and water under stirring. After complete dissolution, 1 mL of ammonia solution was added. After thorough stirring, 0.28 mL of formaldehyde was added. The mixture was stirred for 24 h, then washed with ethanol/water, and dried in an oven at 40 °C for 24 h. A small amount of sulfur powder (mass ratio of polymer to sulfur = 1:10) was added and thoroughly ground. The resulting mixture was then pyrolyzed under nitrogen atmosphere at 600 °C for 2 h with a heating rate of 2 °C/min.

**Characterizations**

Nitrogen sorption analysis was conducted at 77 K using a Micromeritics ASAP 2460

instrument. Scanning electron microscopy (SEM) images were carried out on a HITACHI SU8020 with an accelerating voltage of 5 kV. Transmission electron microscopy (TEM) images were obtained by Talos F200X. The X-ray diffraction (XRD) patterns were measured using a Bruker D8 Advance X-ray diffractometer with monochromatic Cu Kα irradiation. X-ray photoelectron spectroscopy (XPS) measurements were measured on a ESCALAB250 system with a monochromatic X-ray source (Al Kα hv = 1486.6 eV). FT-IR spectra were performed using a Bruker IFS 66 V/S FT-IR spectrometer. ^13^C solid-state NMR (cross polarization magic-angle spinning (CP/MAS)) spectra were carried out on a Bruker Avance 600 MHz spectrometer. The weight loss of the samples was measured using a thermogravimetric analyzer (TGA Q50) in N_2_ with a heating rate of 10°C min^-1^. In situ ATR-SEIRAS spectra was performed on a Bruker Tensor FTIR spectrometer equipped with a MCT detector.

**Electrochemical Measurements**

1. **Material preparation and required instruments**

All electrochemical tests, including cyclic voltammetry (CV), linear sweep voltammetry (LSV), amperometric *I-T* curve, of the materials were conducted using a computer-controlled electrochemical workstation (CHl 760, Shanghai Chenhua Instrument Co., ltd.). All analyses were performed in a three-electrode system: a catalyst-coated rotating ring disk electrode (RRDE, disk area: 0.2475cm^2^ and ring area: 0.1866cm^2^), rotating disk electrode (RDE, area: 0.1975cm^2^), or carbon paper as the working electrode; a graphite rod (for RRDE and RDE) or platinum wire (for carbon paper) as the counter electrode; the Ag/AgCl (saturated KCl solution) as the reference electrode; and Ar-O_2_-saturated 0.1 M KOH solution as the electrolyte. In this work, all potentials were relative to the reversible hydrogen electrode converted by the following equation (E (reversible hydrogen electrode, RHE) = E (Ag/AgCl) + 0.0591 × pH + 0.241 V).

1. **Electrode preparation**

For rotating ring disk electrode (RRDE). The catalyst inks were prepared as follows: 25 mg of prepared catalysts were dispersed in the mixture containing deionized water (770 μL), isopropanol (200 μL) and 5 wt% Nafion solution (30 μL) by ultrasonic oscillation for at least 1 h to form a homogeneous suspension. Then 5 μL of homogeneous ink was dropped on the glassy carbon (GC) disk and dried naturally. The catalyst loading was calculated to be 40.4 ug cm^-2^. The collection efficiency (N) of the RRDE electrode was measured to be 0.37 (see the calibration plot below).


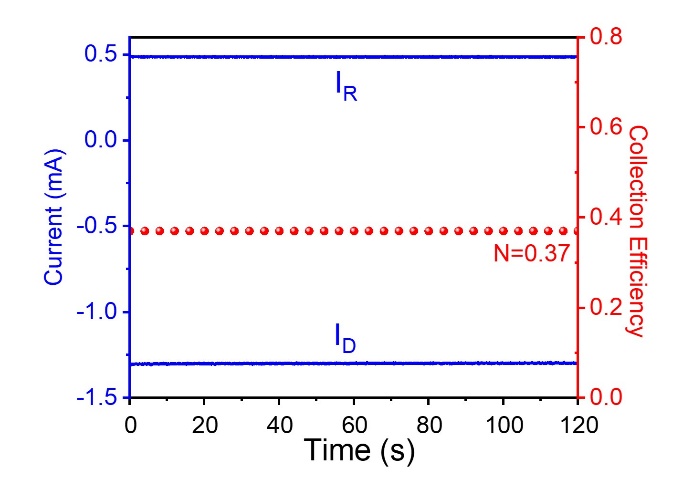


For carbon paper. The catalyst inks were prepared as follows: 25 mg of prepared catalysts were dispersed in the mixture containing deionized water (770 μL), isopropanol (200 μL) and 5 wt% Nafion solution (30 μL) by ultrasonic oscillation for at least 1 h to form a homogeneous suspension. Then 100 μL of homogeneous ink was dropped on the carbon paper and the catalyst-coated surface is 1 cm^2^.

1. **Calculation of H_2_O_2_ selectivity, electron transfer number and Tafel slope**

For RRDE. The catalyst-coated RRDE electrode is equipped as a working electrode, and then the electrochemical workstation performs cyclic voltammetry (CV) and linear sweep voltammetry (LSV), respectively. To evaluate the response to ORR of samples, the CV test was performed in Ar- and O_2_-saturated electrolyte, respectively, at the scan rate of 10 mV/s, with ring voltage turned off. In addition, the LSV test was conducted in O_2_-saturated electrolyte at the scan rate of 5 mV/s, with the ring voltage setting to 0.54 V (*vs*. Ag/AgCl).

The H_2_O_2_ selectivity (H_2_O_2_ %) and the electron transfer number (n) was calculated by **Eq. 1** and **Eq. 2**.

$H_{2}O_{2}\%=200\times\frac{I_{R}}{I_{D} + I_{R}/N}$ (1)

$n=4\times\frac{I_{D}/N}{I_{D} + I_{R}/N}$ (2)

where I_R_ is the ring current, I_D_ is the disk current, and N is the collection efficiency.

The Tafel slope was determined from LSV obtained on RRDE. The Tafel slope was calculated by **Eq. 3**.

$E=a+b\times\log j$ (3)

where E is the potential in V *vs*. RHE, a is the exchange current density, *j* is the current density (mA cm^-2^), and b is the Tafel slope in mV/dec.

1. **H_2_O_2_ yield test**

The H_2_O_2_ production process is performed in a self-assembled flow cell with a three-electrode system. The catalyst was coated homogenously on the gas-diffusion layer (GDL, SGL-39BB) with a loading of 200 μg by using 100 μL ink with the concentration of 2 mg/mL (2.0 mg catalyst powder in the mixture of 770 μL of deionized water, 200 μL isopropanol and 30 μL of 5 wt% Nafion solution). The catalyst loading was calculated to be 200 μg cm^-2^.

For three-electrode tests, the anode and cathode were separated by a pretreated Nafion membrane. 1 M KOH (3 L) as electrolyte was cycled using pumps on each side. The GDL coated with catalyst (active area of 1 × 1 cm^2^), a platinum foil (2 × 2 cm^2^), and Ag/AgCl electrode were used as the working electrode, counter electrode and reference electrode, respectively. Pure O_2_ was purged at 100 mL/min though diffusion channel to the backside of the GDL during the test. And the i-t test was first performed for 10 min at different potentials (-0.4, -0.2, 0, 0.2 and 0.4 V *vs*. RHE). At the end of the test, the electrolyte was collected. The H_2_O_2_ concentration in the electrolyte was tested by the potassium titanium oxalate colorimetric method.

1. **Determination of H_2_O_2_ concentration**

The potassium titanate oxalate colorimetric method was applied to determine the concentration of H_2_O_2_. Specifically, a certain volume of electrolyte was added into a mixture of 0.05 M potassium titanium oxalate K_2_TiO(C_2_O_4_)_2_ solution (0.5 mL), 3 M H_2_SO_4_ solution (0.5 mL) and ultrapure water to ensure the total volume of solution is 5 mL. Next, the ultraviolet-visible (UV-vis) spectroscopy (Taisitefx UV-1802G UV-visible spectrophotometer) was used to determine the absorbance at 400 nm and calculate the content of the H_2_O_2_ in the samples by comparing with the standard curve. The Faradaic Efficiency (FE) of H_2_O_2_ generating could be calculated by **Eq. 4**.

$FE \left( \% \right)=100\times\frac{2\times C\times V\times F}{Q}\times100\%$ (4)

where C is the concentration of H_2_O_2_ (mol L^-1^), V is the electrolyte volume (L), F is the faraday constant (96485C mol^-1^), and Q is the passed charge amount (C).

1. **In situ ATR-SEIRAS measurements**

In situ ATR-SEIRAS spectra was performed on a Bruker Tensor FTIR spectrometer equipped with a MCT detector. The electrochemical test was conducted in a custom-made three electrode electrochemical single cell. A Pt wire (commercial) and a saturated Ag/AgCl were used as the counter and reference electrodes, respectively. The carbon paper (0.5×0.5 cm^2^) was loaded with 0.1 mg electrocatalyst and then served as the working electrode. The in-situ ATR-SEIRAS spectrum were recorded by varying the potential stepwise from 0.5 V to 0 V *vs*. RHE in O_2_-saturated 0.1 M KOH and run time was 60 s.

**Computational details**

All spin-polarized calculations presented were performed using the Vienna Ab Initio Simulation Package (VASP). The electron-ion interactions were described by the Projector Augmented Wave (PAW) method. The exchange-correlation energy of electrons was treated within the Generalized Gradient Approximation (GGA), employing the Perdew-Burke-Ernzerhof (PBE) functional form. Default PAW pseudopotentials were adopted for S, N, O, C, and H. The plane-wave basis set energy cutoff was set to 450 eV. A Gaussian smearing width of σ = 0.2 eV was applied based on the Methfessel-Paxton technique, and Monkhorst-Pack k-point sampling was utilized. For structural optimization of the unit cell, a k-point mesh of 7×7×7 was employed, whereas a 3×3×1 k-point mesh was used for geometric optimization of surfaces. The convergence criteria for energy and forces were set to 1×10⁻⁴ eV and 0.03 eV/Å, respectively.

The binding energy (*E*_b_) of the screened surfaces was calculated using Eq. (1).

*E*_b_ = *E*_slab_ - *E*_def_ - *E*_M_ (1)

Where *E*_slab_ is the total energy of the doped surface, *E*_def_ is the total energy of the defective graphene excluding doped atoms, and *E*_M_ is the energy of the doped atoms.

Since the reaction temperature is room temperature (25 ℃), the energies of gaseous reactants and products have been corrected using the calculation formula shown in Eq. (2).

$\Delta G=\Delta E+\Delta E_{ZPE}+\Delta H-T\Delta S+Rln(\frac{P}{P^{0}})$ (2)

Δ*E* refers to the reaction energy directly obtained from Density Functional Theory (DFT) calculations; Δ*E*_ZPE_ denotes the zero-point energy change; Δ*S* represents the entropy change; *T* is the system temperature, which is 298.15 K in this study; Δ*H* stands for the enthalpy change; *P* is the actual pressure; and *P*_0_ is the standard pressure.

**Finite element analysis simulations**

The numerical simulations were performed using COMSOL Multiphysics 6.3 to investigate the influence of catalyst morphology on the electrolyte flow field and the concentration of oxygen at the catalyst surface. A two-dimensional model of a microfluidic channel was established. The flow of an incompressible, Newtonian electrolyte was described by the steady-state Navier-Stokes equations for laminar flow, while the transport and consumption of dissolved oxygen were modeled using the convection-diffusion equation. The governing equations for fluid flow are the continuity and momentum conservation equations:


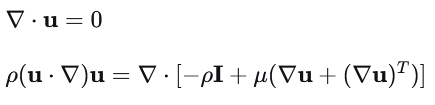


where ρ is the density, u is the velocity vector, p is the pressure, and μ is the dynamic viscosity. The distribution of dissolved oxygen was solved through the following mass transport equation:


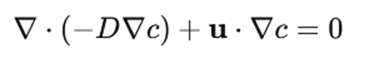


where c is the concentration of dissolved oxygen and D is its diffusion coefficient in the electrolyte. For the boundary conditions, the channel inlet was set to a laminar inflow with a constant pressure, and the outlet was set to a zero-pressure condition. No-slip conditions were applied at all walls. For the mass transport, a fixed oxygen concentration was defined at the inlet, and a convective flux was set at the outlet. The electrochemical reduction of oxygen to hydrogen peroxide on the catalyst surface was modeled as a first-order reaction, implemented as a flux boundary condition.


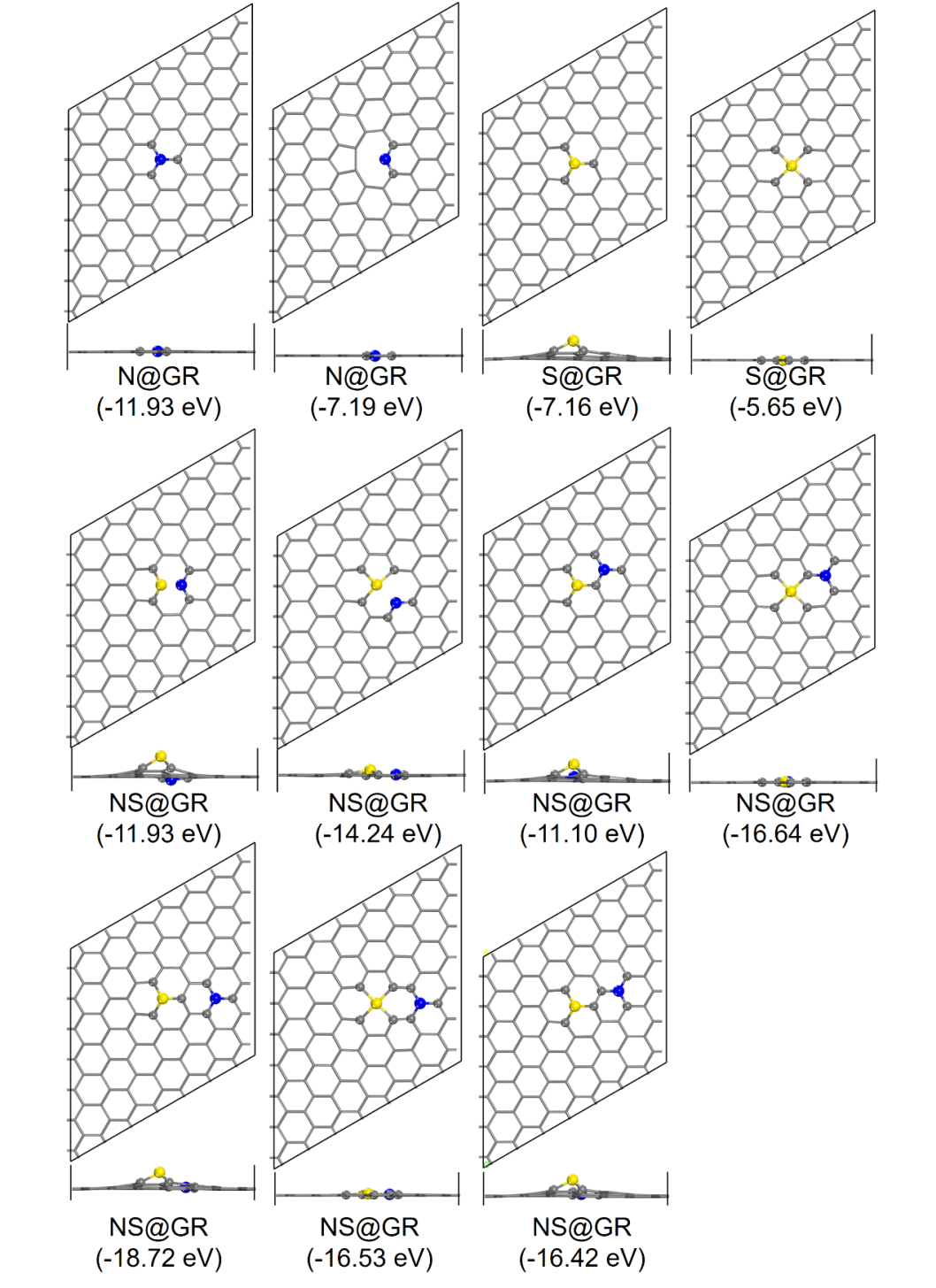


**Figure S1**. Atomic models of N-doped (N@GR), S-doped (S@GR), and N,S-dual-doped (NS@GR) graphene with different coordination configurations, and the corresponding binding energies of heteroatoms. Color code: gray, C; blue, N; yellow, S.


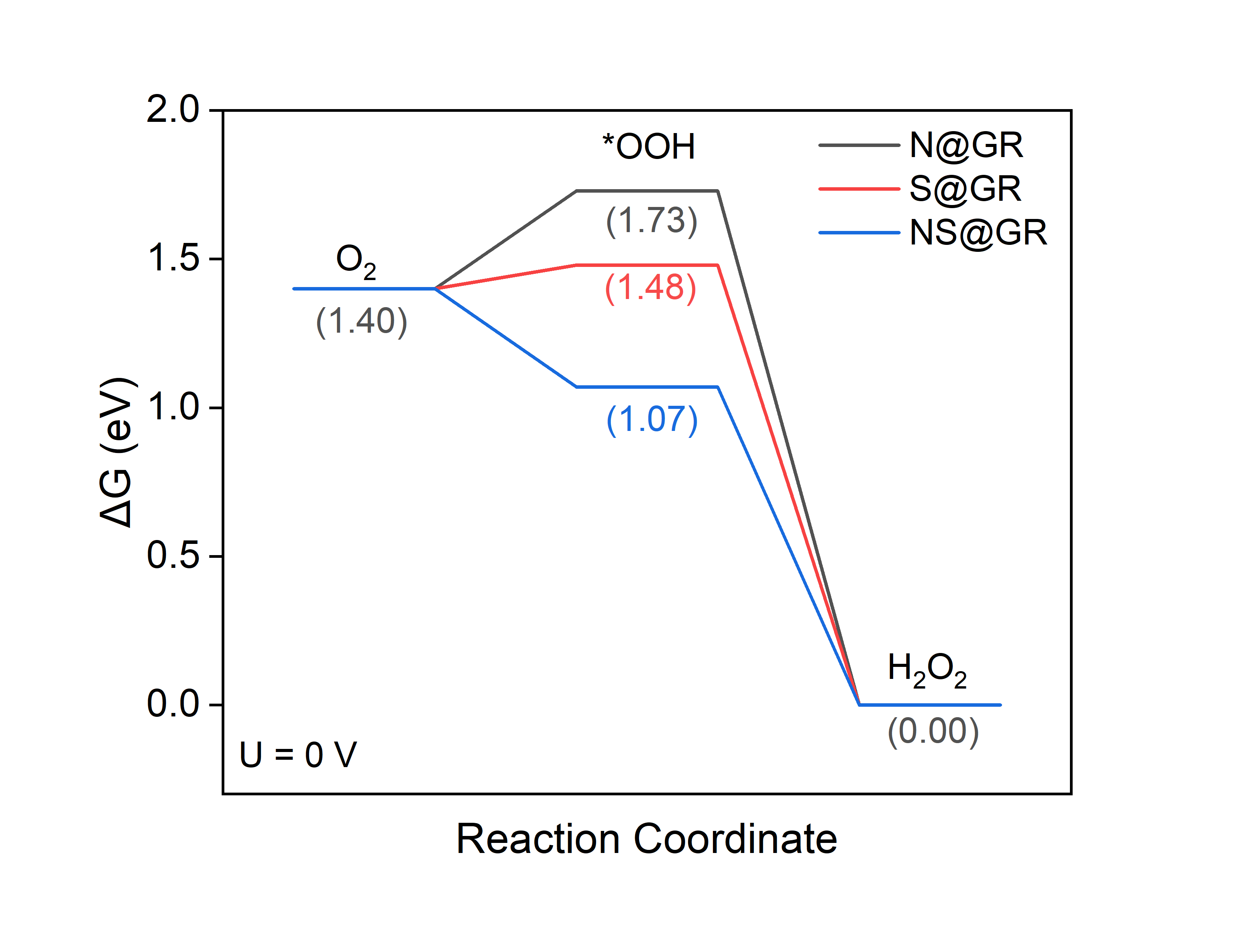


**Figure S2**. Gibbs free energy diagrams of the 2e⁻ ORR pathways on the three models of N@GR, S@GR, and NS@GR at 0 V. The NS@GR model exhibits the lowest overpotential for H_2_O_2_ formation.


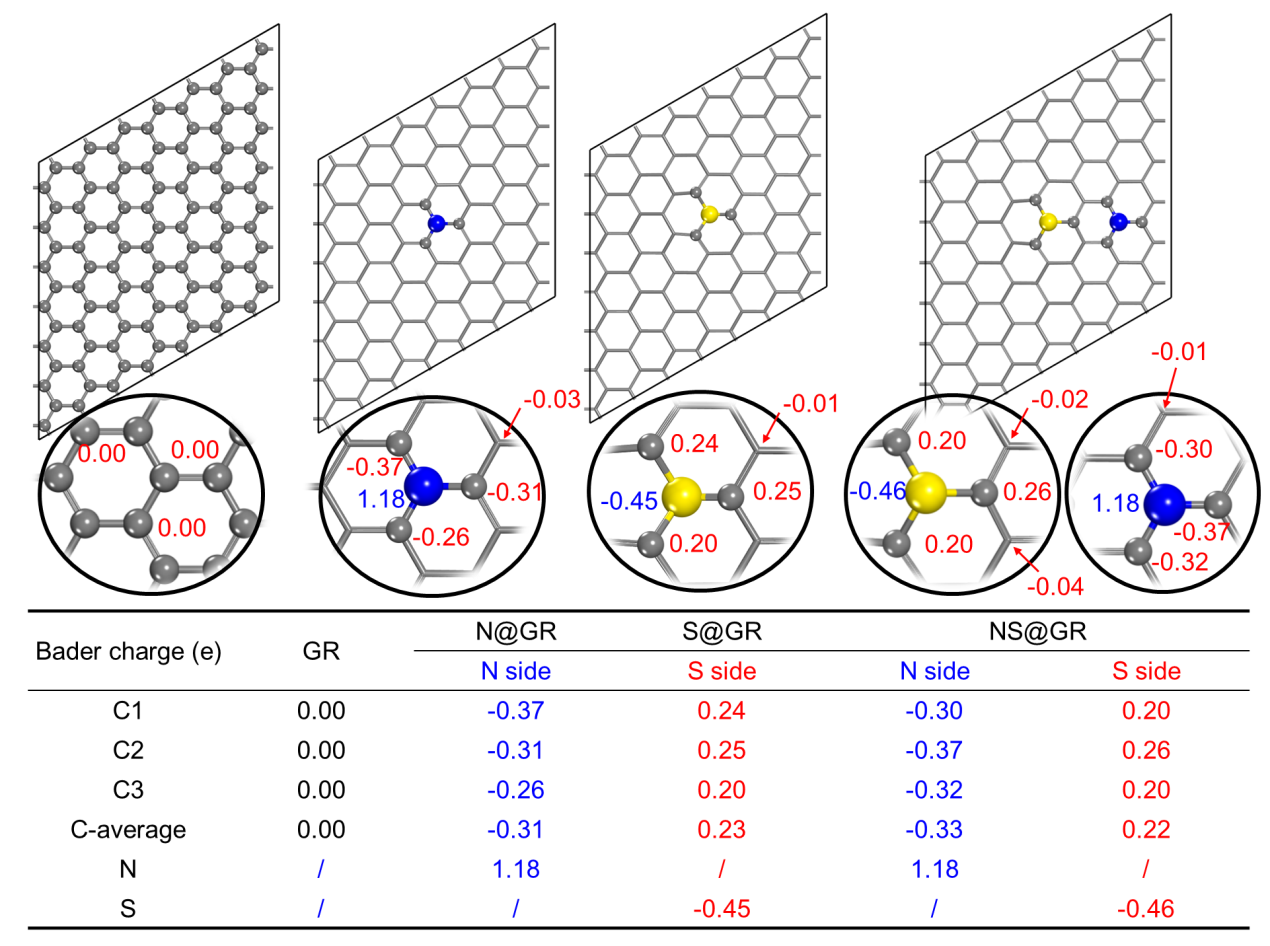


**Figure S3**. Bader charge analysis of pristine graphene GR, N@GR, S@GR, and NS@GR. The insets show the atomic structures with charge values (in |e|) labeled on selected atoms. The color scale indicates electron density redistribution.


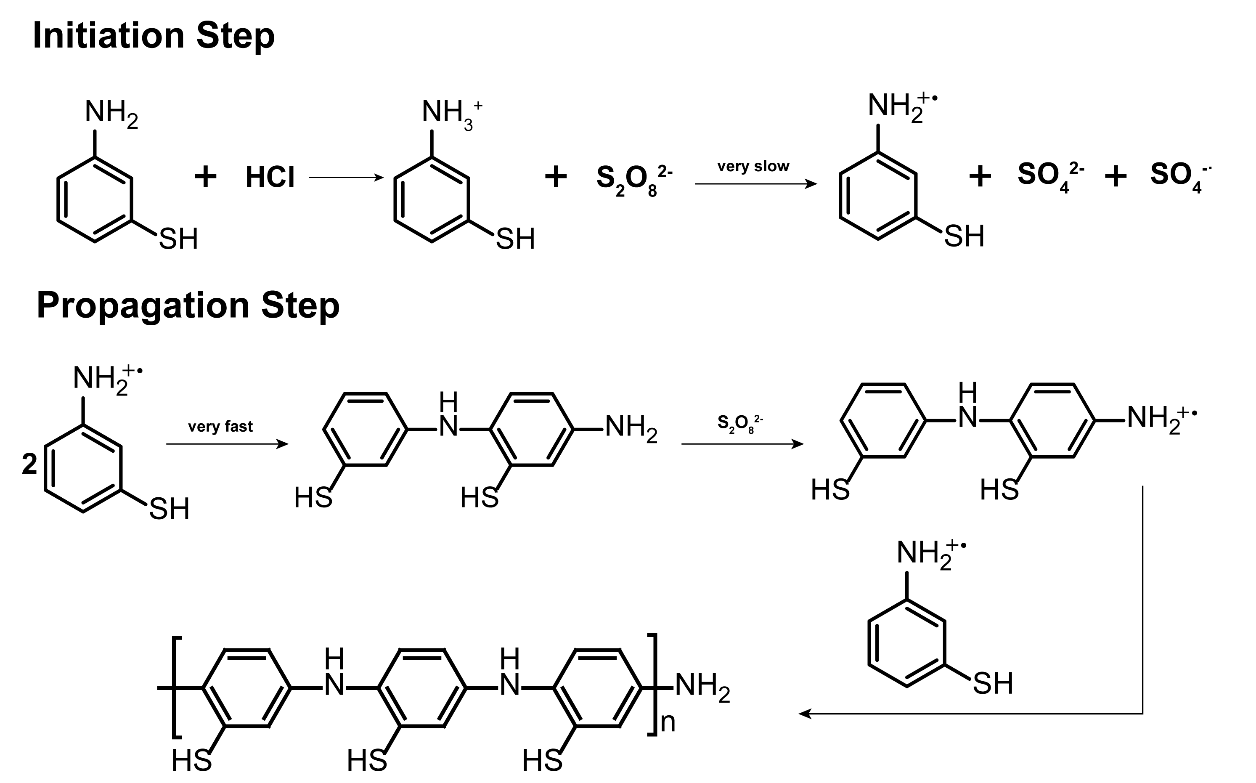


**Figure S4**. Proposed schematic illustration of the chemical oxidative polymerization mechanism of the ATP monomer.


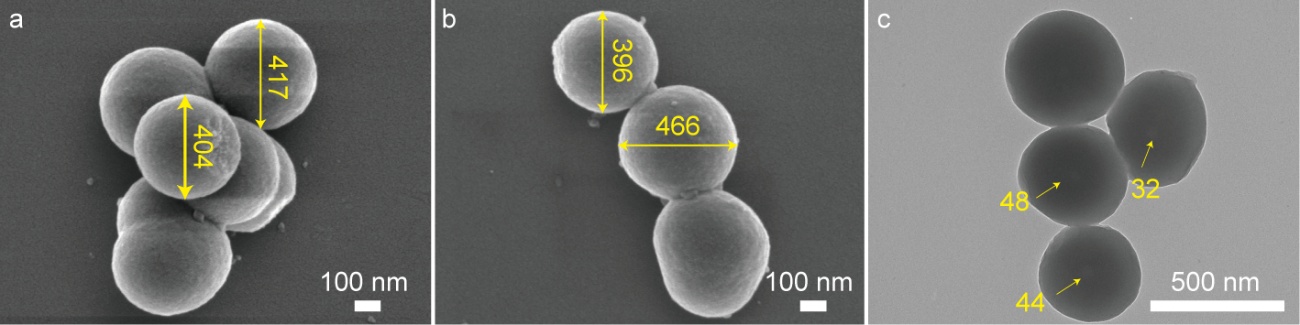


**Figure S5**. (a-b) The external diameter of MPPs-0. (c) The internal diameter of MPPs-0.


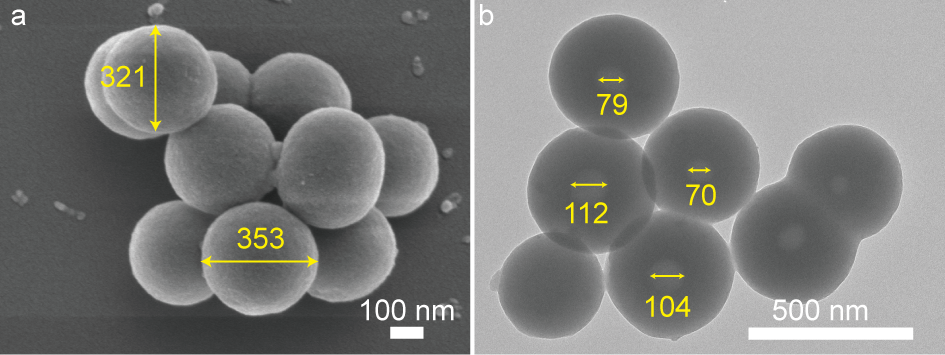


**Figure S6**. (a) The external diameter of MPPs-0.1. (b) The internal diameter of MPPs-0.1.


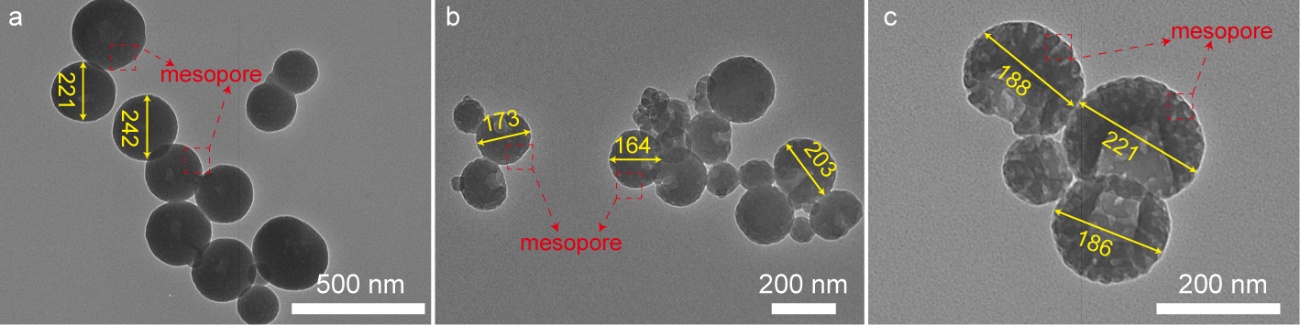


**Figure S7**. TEM images of (a) MPPs-0.2, (b) MPPs-0.3 and (c) MPPs-0.4. The corresponding particle size and representative mesoporous structural regions have been marked in the images.


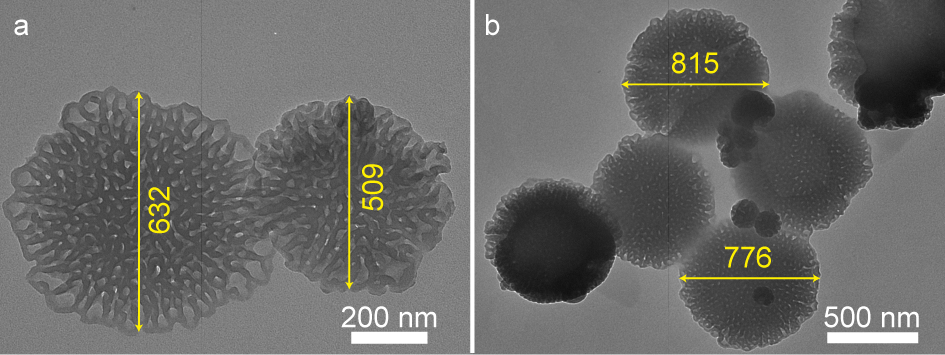


**Figure S8**. TEM images of (a) MPPs-0.8 and (b) MPPs-1.5. The sizes of the corresponding nanosheet have been given in the images.


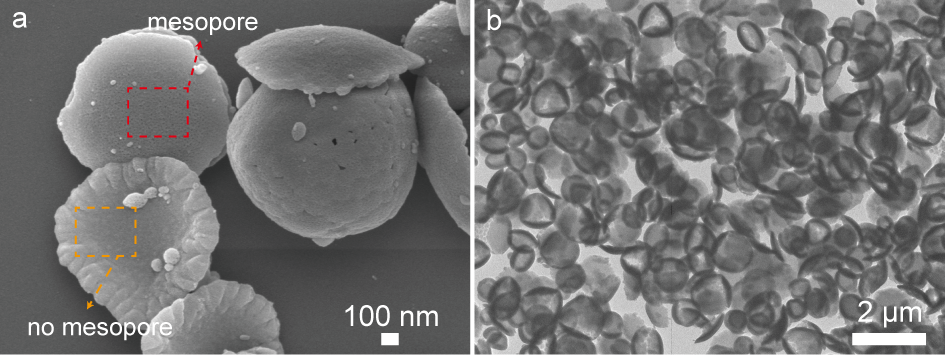


**Figure S9**. (a) SEM images of the corresponding structure of the concave and convex surfaces of MPPs-2.0. (b) TEM images of MPPs-2.0 over a large area.


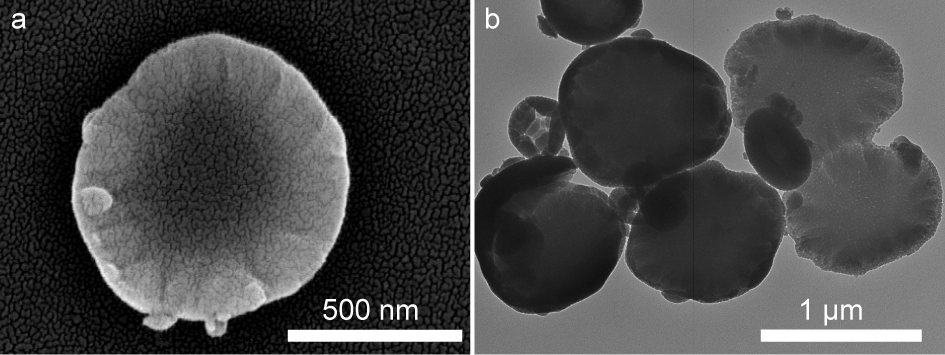


**Figure S10**. SEM (a) and TEM (b) images of MPPs-3.0.


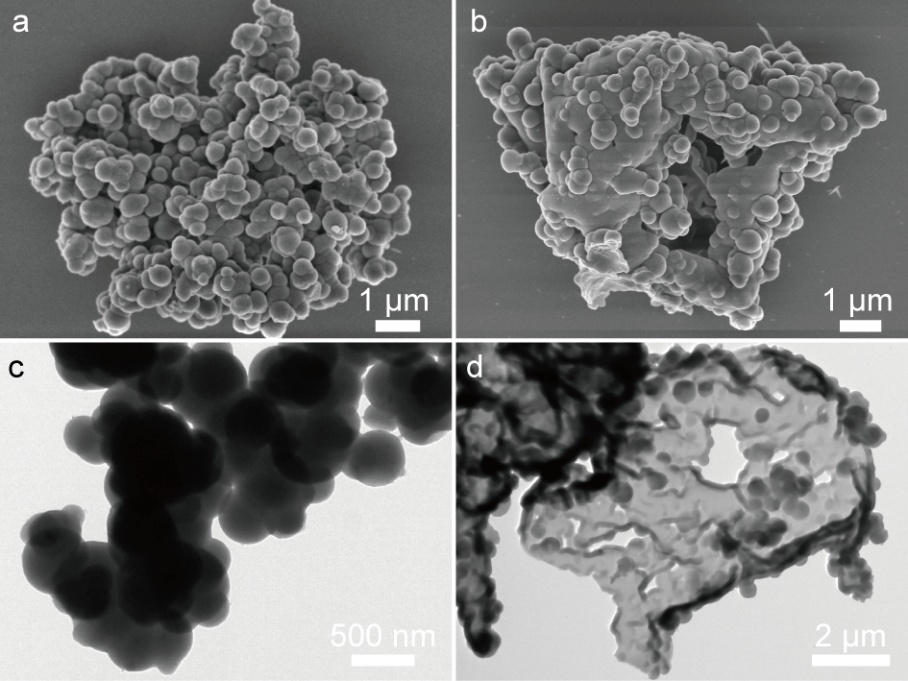


**Figure S11**. SEM and TEM images of samples prepared in the absence of both F127 and TMB (a, c). SEM and TEM images of samples prepared by only introducing TMB (b, d).


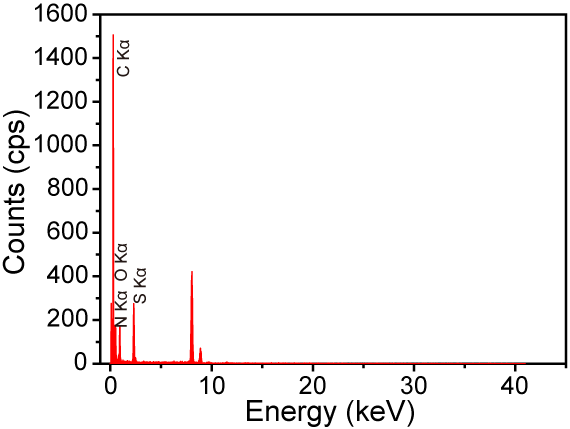


**Figure S12**. EDS element distribution spectrometry.


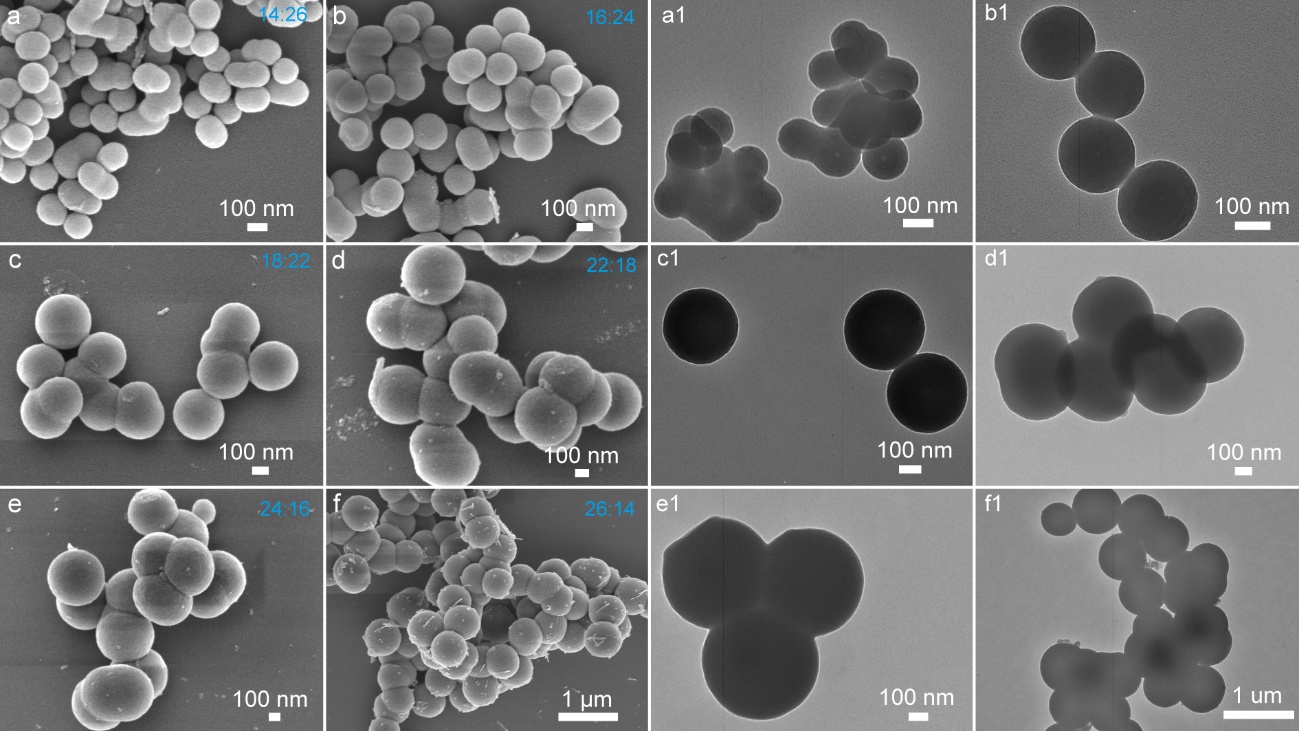


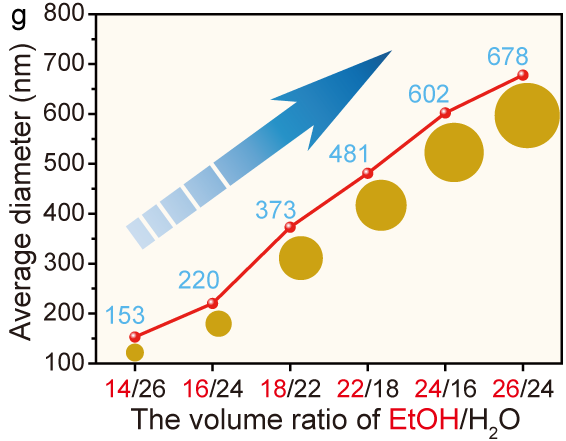


**Figure S13**. SEM (a-f) and TEM (a_1_-f_1_) images of samples prepared with variations in ethanol content. (g) Curve of submicron spheres size with ethanol volume fraction.

**
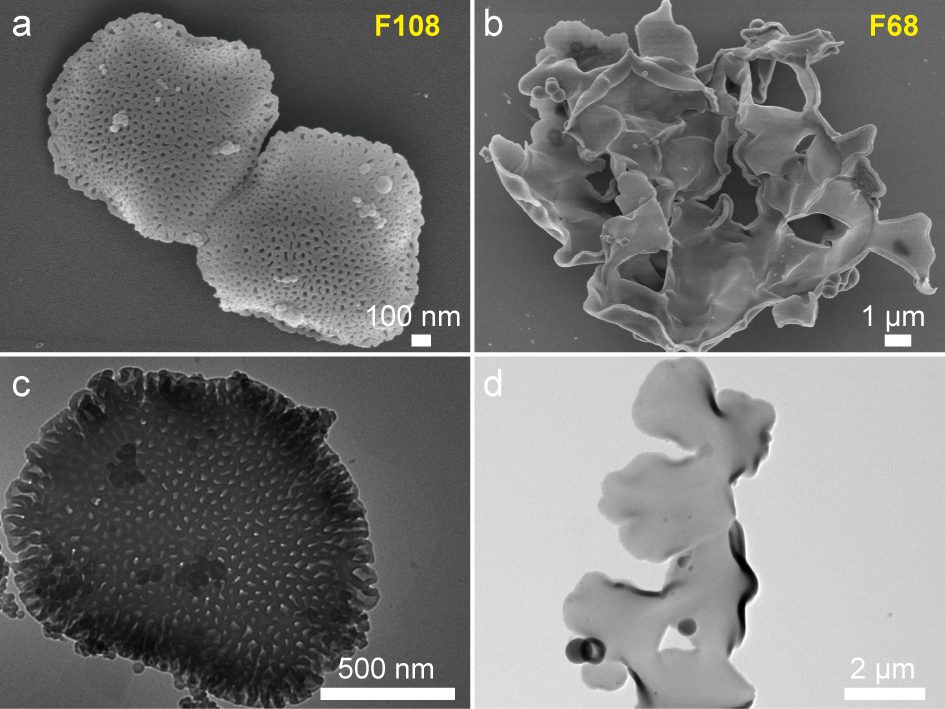
**

**Figure S14**. SEM and TEM images of samples prepared by using F108 (a, c) and F68 (b, d) as structure-directing agent, respectively. (Note: Both F108 and F68 were used in 0.8 g, TMB was 0.8 mL)


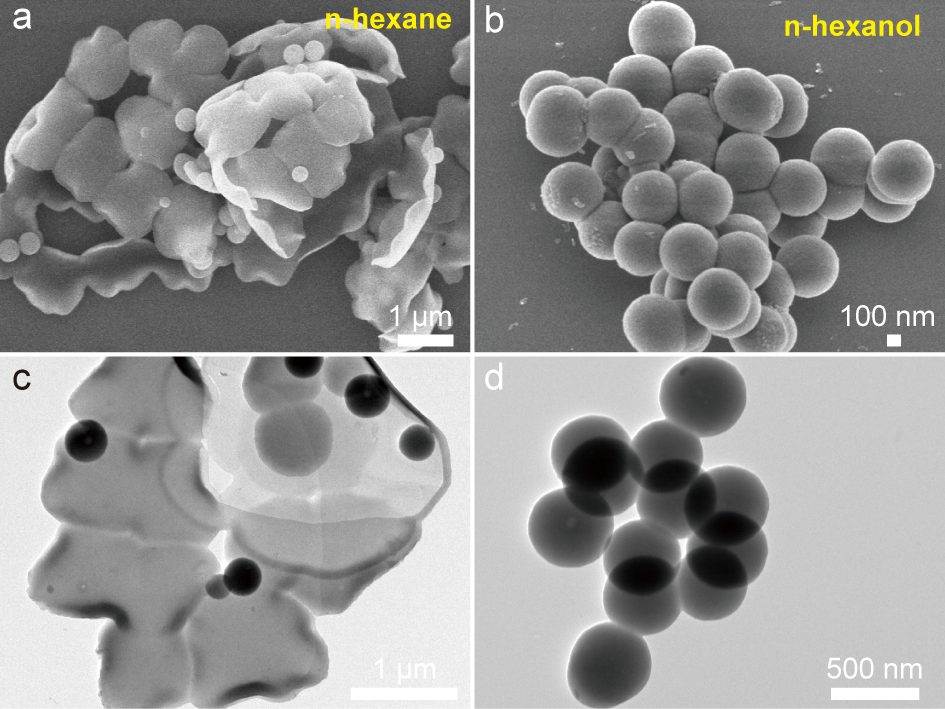


**Figure S15**. SEM and TEM images of samples prepared by using n-hexane (a, c) and n-hexanol (b, d) as oil phase. (Note: Both n-hexane and n-hexanol were used in 0.8 mL)


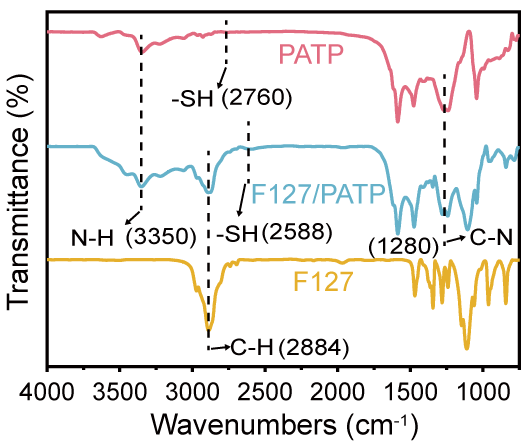


**Figure S16**. FT-IR spectrum of PATP, F127/PATP and F127.


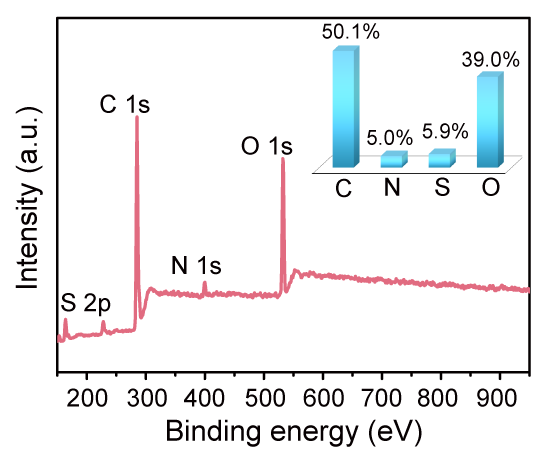


**Figure S17.** XPS survey spectrum and the element mass percentages of C, N, S and O.


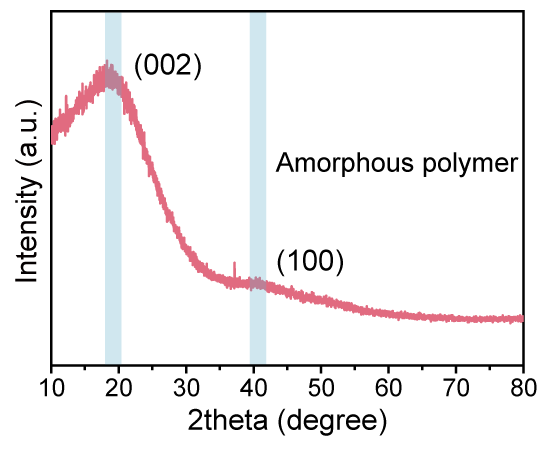


**Figure S18.** XRD pattern.


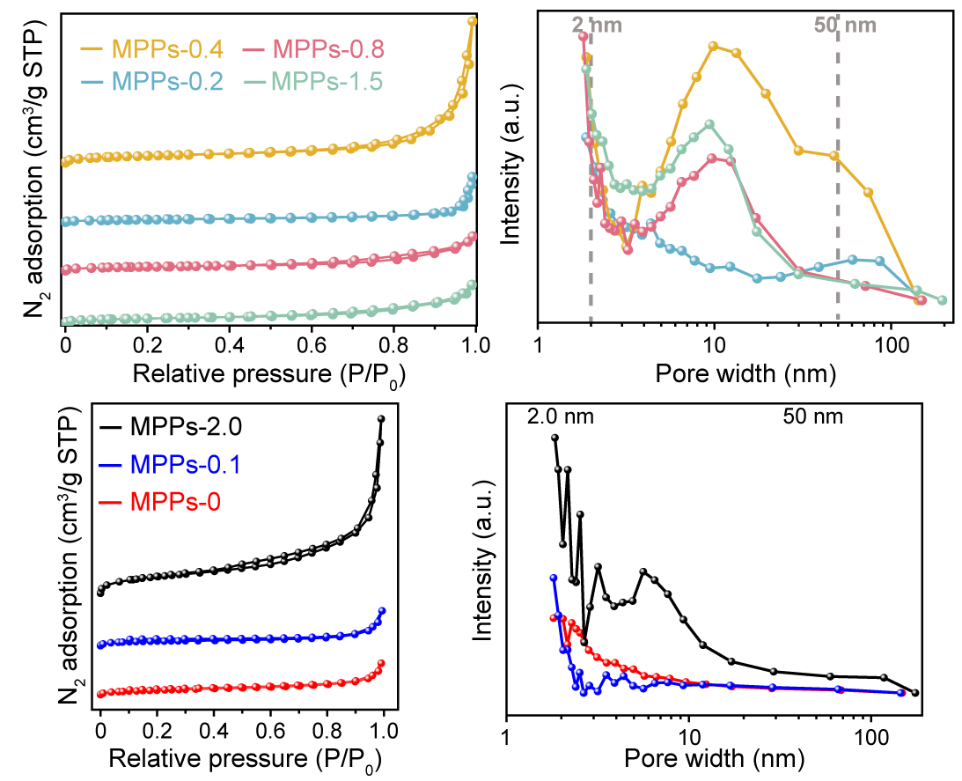


**Figure S19**. Nitrogen adsorption-desorption isotherms and pore size distribution.


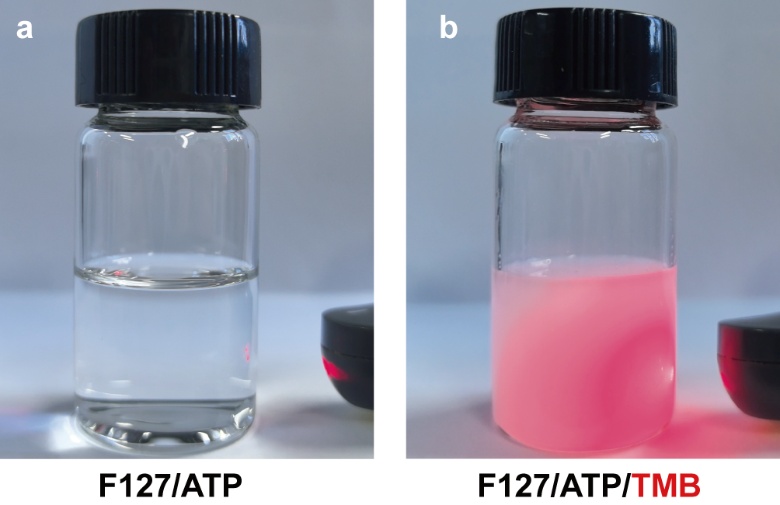


**Figure S20**. Optical photographs: (a) F127 and ATP are dissolved in a water /ethanol mixture. (b) F127, ATP and TMB are dissolved in a water /ethanol mixture. (Note: Compared with conventional micelle system, the F127/ATP/TMB system shows an obvious Tyndall phenomenon because numerous TMB droplets existed in the system.)


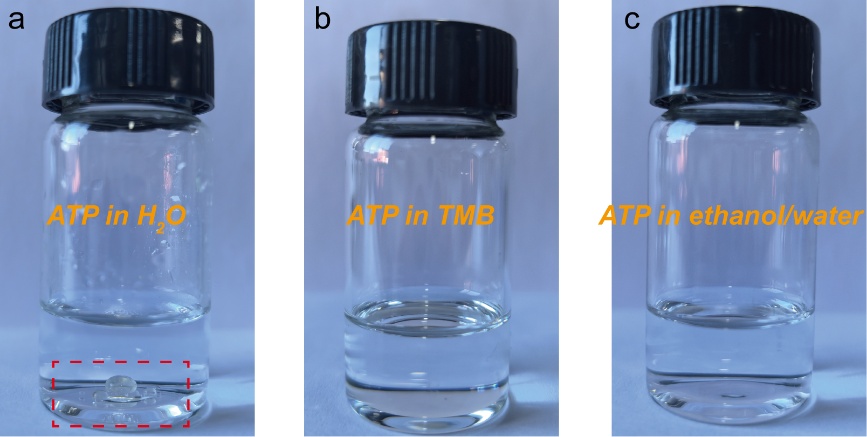


**Figure S21**. Optical photographs of ATP monomer dissolved in different liquid phases. ATP shows better affinity for the oil phase (TMB) than for water or ethanol.


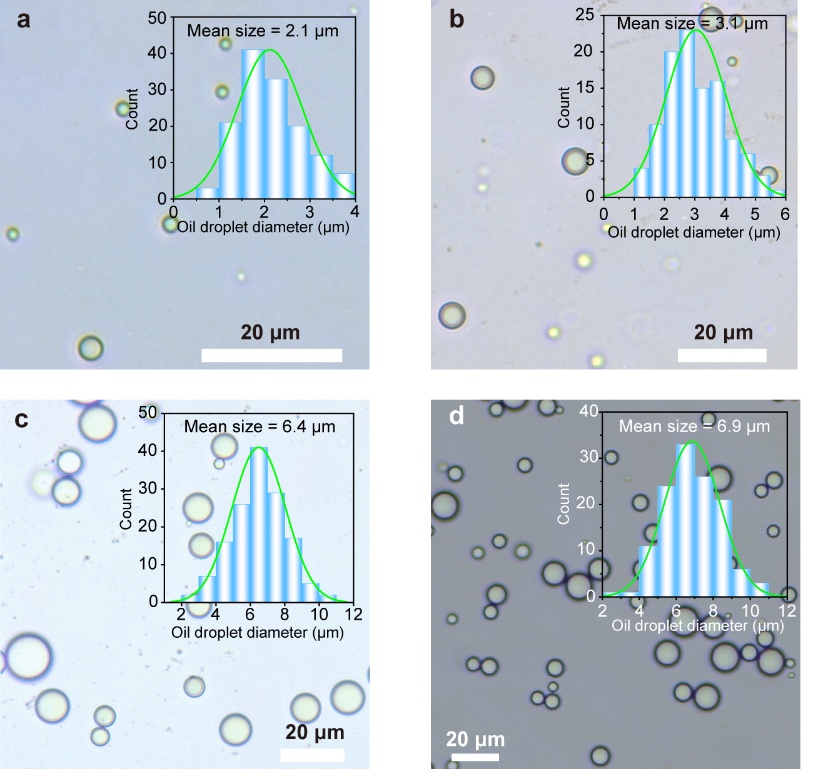


**Figure S22**. Optical micrographs of F127-stabilized TMB oil droplets with TMB volumes of (a) 0.1 mL, (b) 0.4 mL, (c) 1.5 mL, and (d) 2.0 mL.


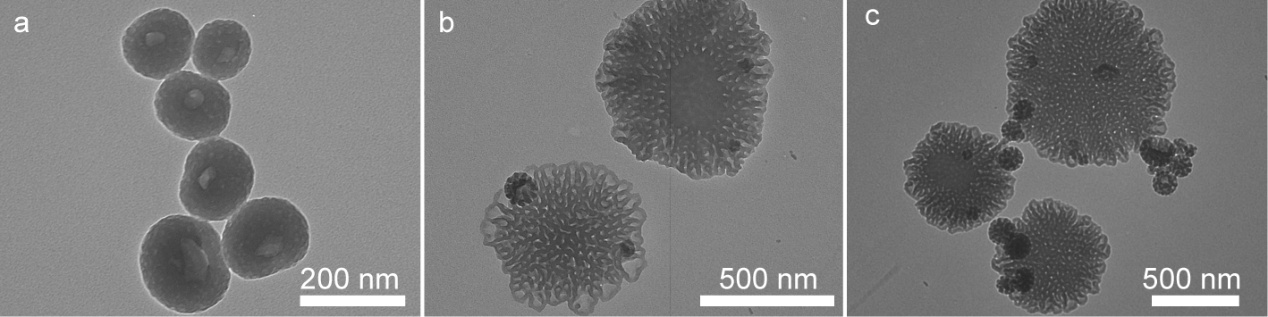


**Figure S23**. TEM images (a-c) of MCPs-0.2, MCPs-0.8 and MCPs-1.5, respectively.


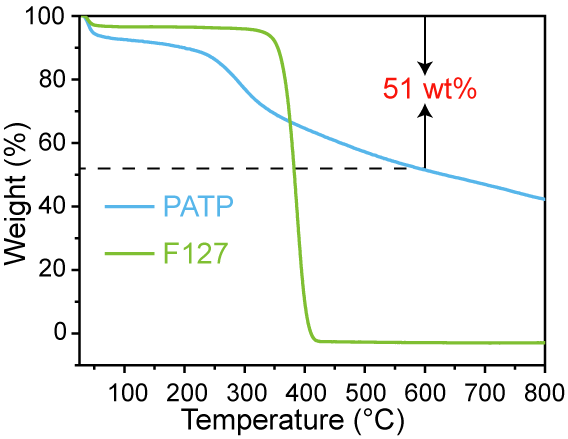


**Figure S24**. TGA curves of PATP and F127 under N_2_ atmosphere with a ramp rate of 10℃ min^-1^. (Note: The weight loss at 350-415 °C corresponded to F127, suggesting that F127 played a crucial role in the self-assembly process.)


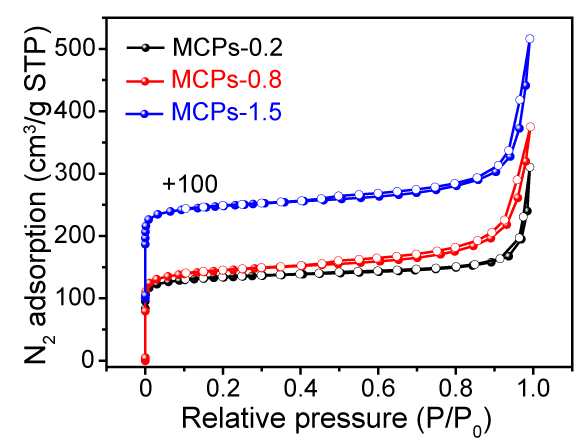


**Figure S25**. N_2_ adsorption–desorption isotherms of MCPs-0.2, MCPs-0.8, and MCPs-1.5. All samples show a combined Type *I/IV* isotherm with an H3 hysteresis loop, indicating the presence of both micropores and mesopores.


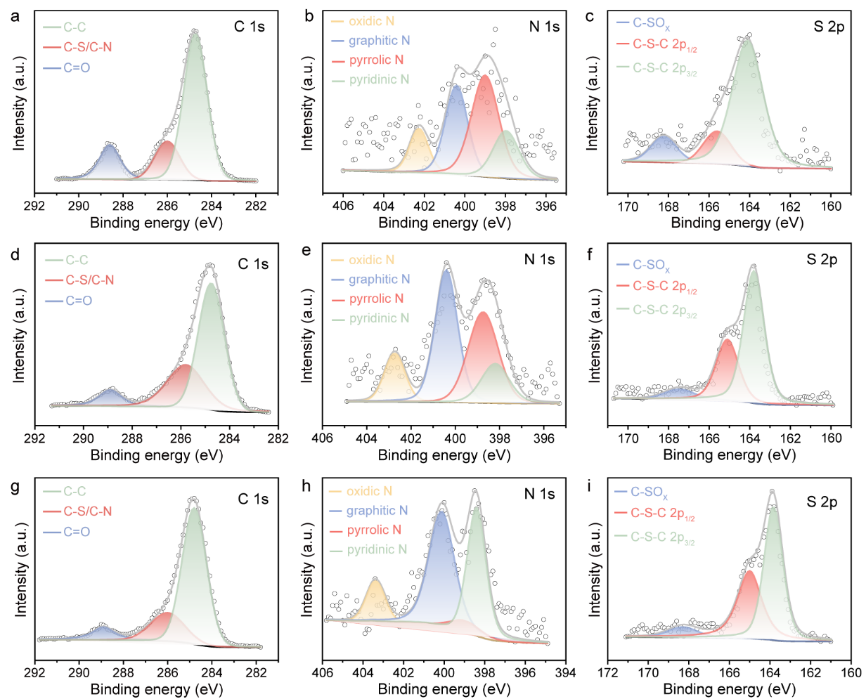


**Figure S26**. XPS survey spectrum and high-resolution XPS spectra of C 1s, N 1s, and S 2p, respectively. (a-a3) MCPs-0.2, (b-b3) MCPs-0.8, (c-c3) MCPs-1.5. (600 °C)


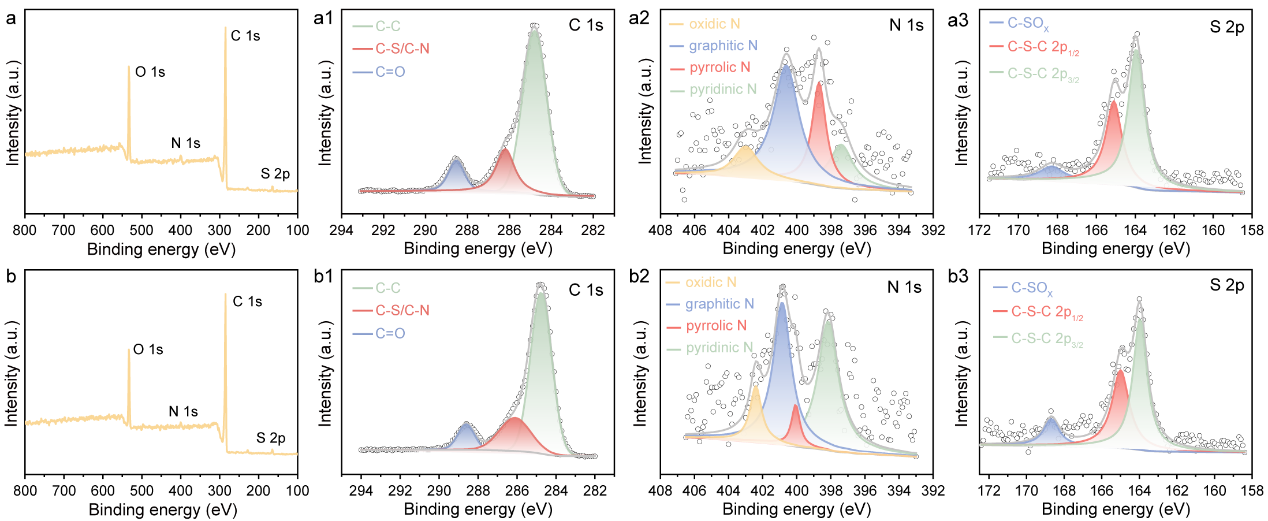


**Figure S27**. The XPS results corresponding to the samples MPPs-0.2 after being calcined at different temperatures, respectively. (a-a3) 700 °C, (b-b3) 800 °C.


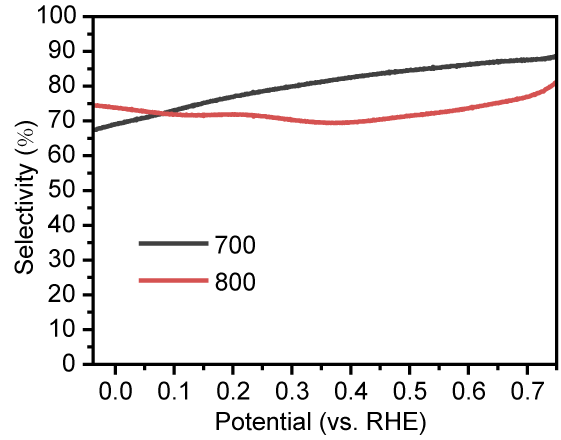


**Figure S28**. H_2_O_2_ selectivity of MCPs-0.2 samples pyrolyzed at 700 °C and 800 °C. The selectivity decreases with increasing pyrolysis temperature due to reduced N, S content.


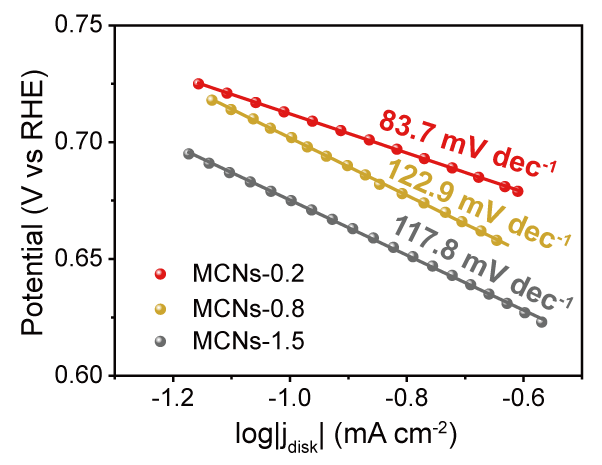


**Figure S29**. Tafel plots derived from LSV curves.


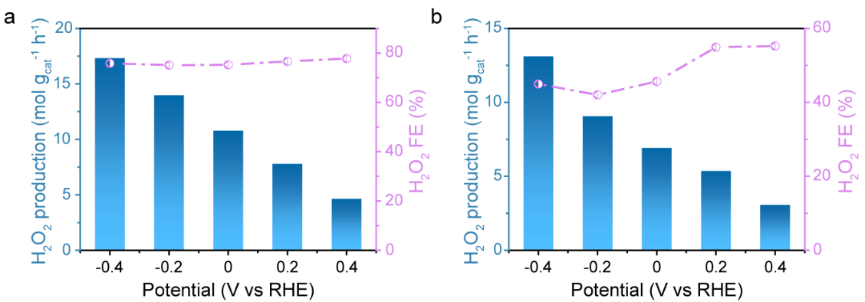


**Figure S30**. H_2_O_2_ production rates and FEs at different potentials. (a) MCPs-0.8, (b) MCPs-1.5.


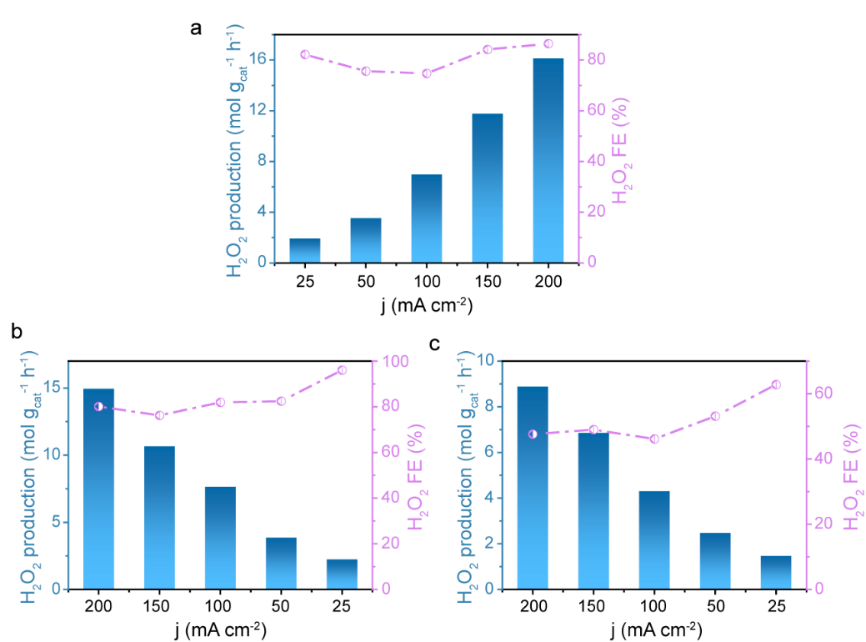


**Figure S31**. H_2_O_2_ production rates and FEs at different current densities. (a) MCPs-0.2, (b) MCPs-0.8, (c) MCPs-1.5.


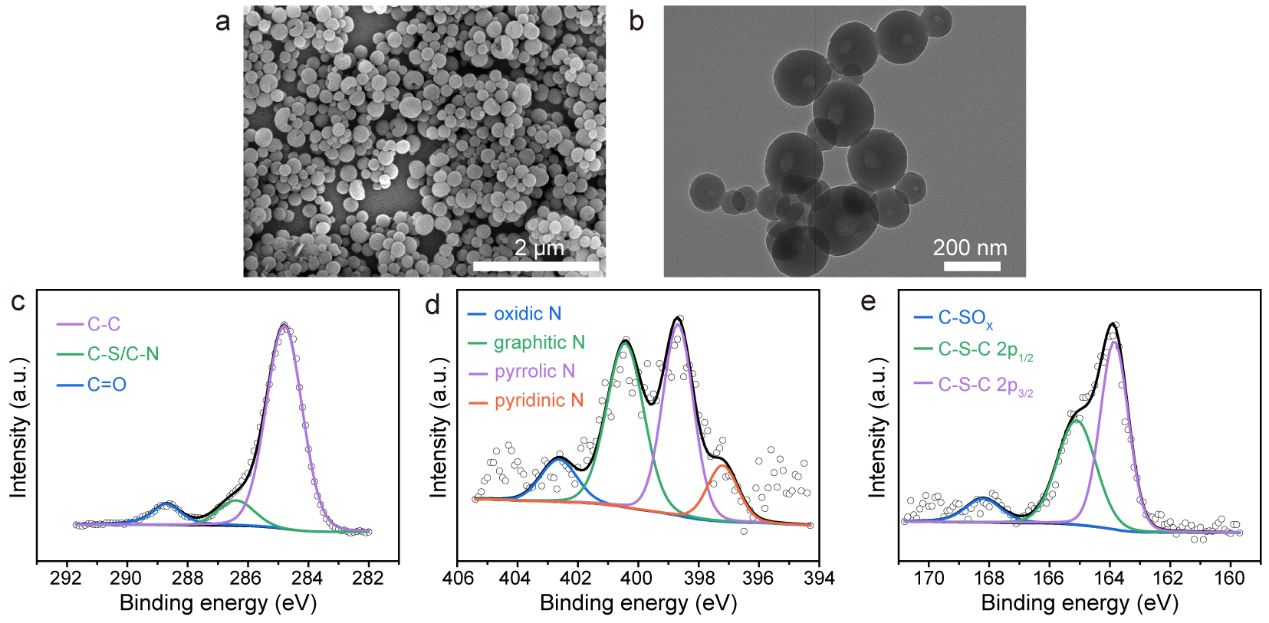


**Figure S32**. Post-stability characterization of MCPs-0.2 after 50 h of electrolysis. (a) SEM image, (b) TEM image, and (c–e) high-resolution XPS spectra of C 1s, N 1s, and S 2p, respectively. No significant changes in morphology, pore structure, or surface composition are observed, confirming excellent durability.


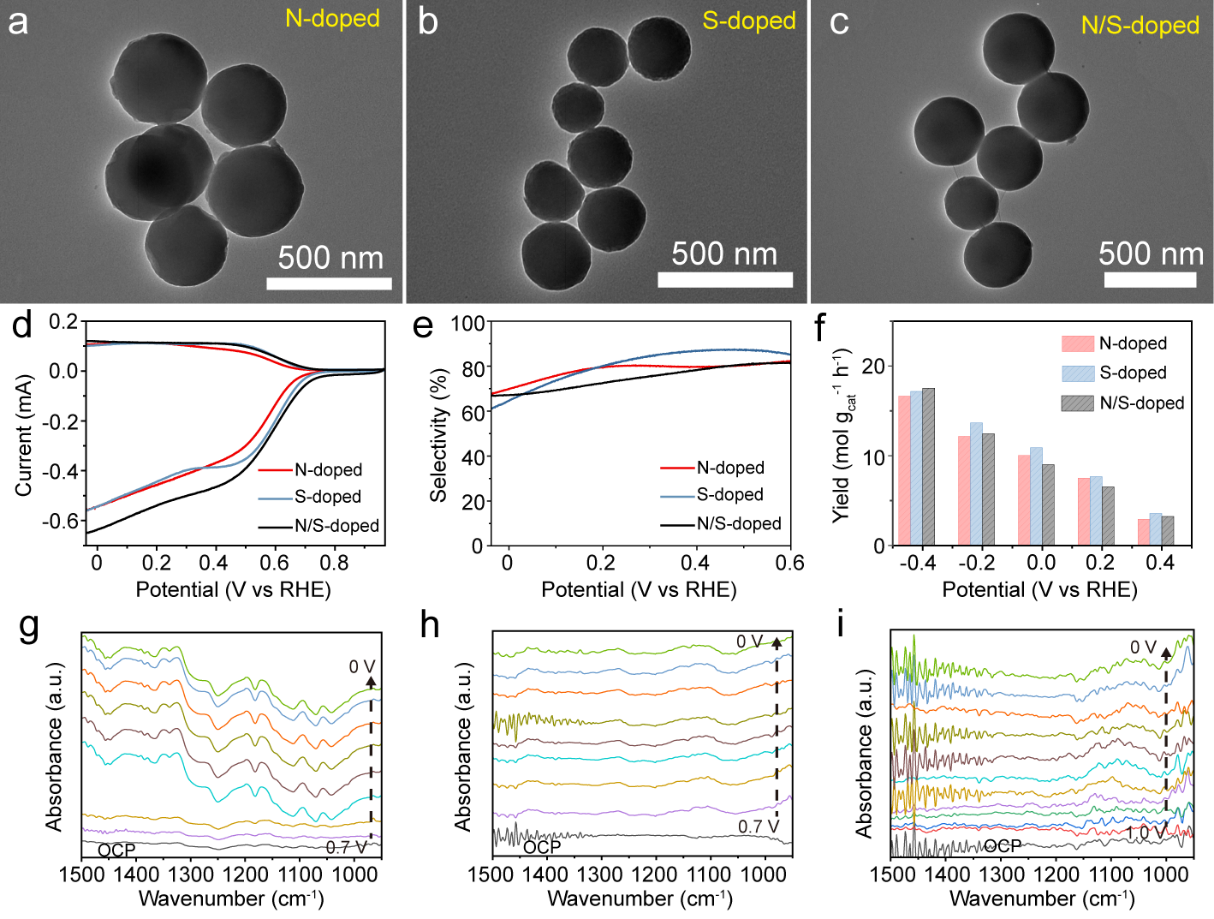


**Figure S33**. (a-c) TEM images of N-doped carbon, S-doped carbon and post-treated N,S-dual-doped carbon. (d) LSV curves, (e) H_2_O_2_ selectivity, (f) H_2_O_2_ production rates at different potentials, (g-i) In situ ATR-SEIRAS spectra for samples. Compared with MCPs-0.2, all control samples show lower selectivity and weaker intermediate signals, highlighting the advantage of in situ molecular-level N,S-dual-doping.


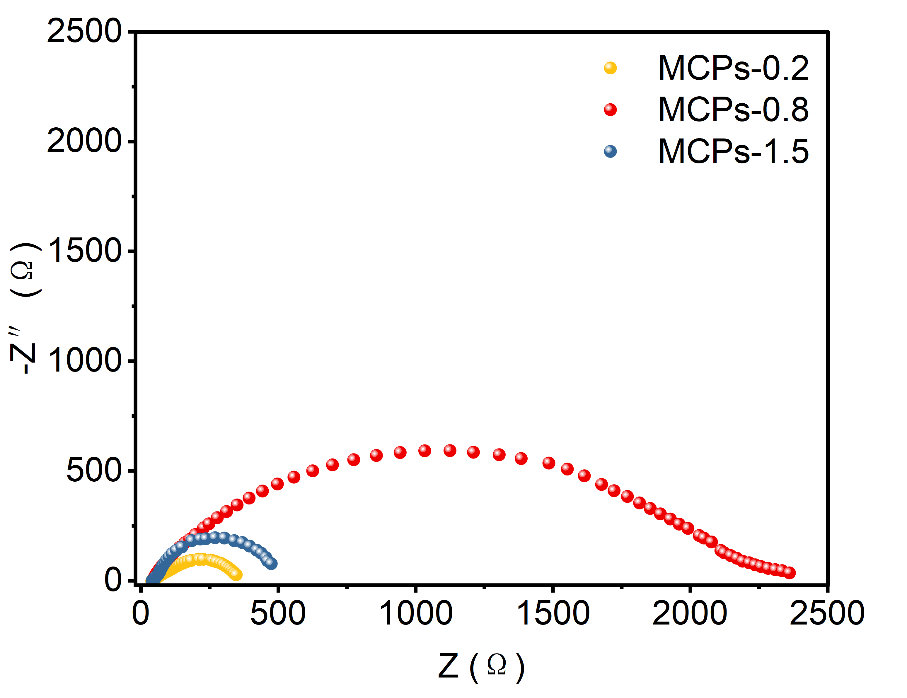


**Figure S34**. EIS plots of MCPs-0.2, MCPs-0.8 and MCPs-1.5.


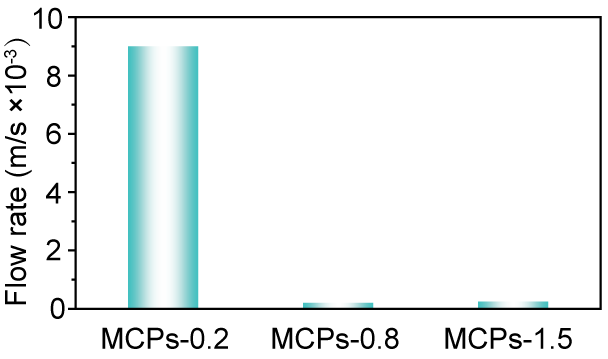


**Figure S35**. The simulation results depicting the flow rate.


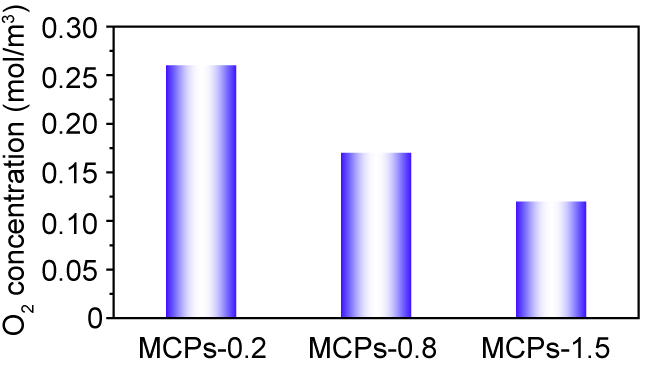


**Figure S36**. The simulation results depicting distribution of O_2_ concentration.

**Table S1**. The Bader charges of the different elements in the N@GR, S@GR, and NS@GR models with and without OOH adsorption, as well as the corresponding Bader charge differences for OOH adsorption (Δ*q* = *q*_*OOH_ - *q*_slab_).

| **Bader charge (e)** | | ***q*_C_** | ***q*_N_** | ***q*_S_** | ***q*_O1_** | ***q*_O2_** | ***q*_H_** |
| --- | --- | --- | --- | --- | --- | --- | --- |
| N@GR | slab | -0.31 | 1.18 | / | / | / | / |
|  | *OOH | -0.62 | 1.16 | / | 0.45 | 0.66 | -0.64 |
|  | Δ*q* | **-0.31** | **-0.02** | / | / | / | / |
| S@GR | slab | / | / | -0.45 | / | / | / |
|  | *OOH | / | / | -0.64 | 0.42 | 0.60 | -0.59 |
|  | Δ*q* | / | / | **-0.19** | / | / | / |
| NS@GR | slab | / | 1.18 | -0.46 | / | / | / |
|  | *OOH | / | 1.20 | -0.72 | 0.51 | 0.64 | -0.55 |
|  | Δ*q* | / | **0.02** | **-0.26** | / | / | / |

Noted: the *q*_C_ is the Bader charge of the active C atom bonded with the OOH species.

**Table S2.** Related structural parameters of MPPs.

| **Samples** | **BET (m^2^/g)** | **Pore volume (cm^3^/g)** | **Pore size (nm)** |
| --- | --- | --- | --- |
| MPPs-0 | 11.6 | 0.032 | 2.2 |
| MPPs-0.1 | 11.5 | 0.031 | 2.5 |
| MPPs-0.2 | 31.9 | 0.239 | 4.4 |
| MPPs-0.4 | 90.9 | 0.751 | 9.8 |
| MPPs-0.8 | 48.6 | 0.181 | 9.8-12.3 |
| MPPs-1.5 | 55.9 | 0.207 | 9.3 |
| MPPs-2.0 | 41.9 | 0.175 | 6.0 |

**Table S3.** Related structural parameters of MCPs at 600 °C.

| **Samples** | **BET (m^2^/g)** | **Pore volume (V, cm^3^/g)** | **V%_micropore_** | **V%_mesopore_** |
| --- | --- | --- | --- | --- |
| MCPs-0.2 | 519.7 | 0.28 | 40.46 | 23.56 |
| MCPs-0.8 | 556.2 | 0.38 | 34.06 | 65.94 |
| MCPs-1.5 | 569.2 | 0.44 | 31.33 | 44.95 |

Note: Percentages are based on total pore volume.

The results clearly demonstrate that all samples possess significant mesopore volumes. And the materials are genuinely hierarchical, with both pore regimes contributing substantially to the total pore volume.

**Table S4.** Related elements content of the samples at different pyrolysis temperatures.

| **Samples** | **C (%)** | **N (%)** | **S (%)** | **O (%)** |
| --- | --- | --- | --- | --- |
| MCPs-0.2-600 | 70.84 | 3.96 | 2.87 | 22.33 |
| MCPs-0.8-600 | 76.80 | 7.22 | 3.36 | 12.62 |
| MCPs-1.5-600 | 76.52 | 7.58 | 4.48 | 11.42 |
| MCPs-0.2-700 | 76.73 | 3.37 | 1.69 | 18.21 |
| MCPs-0.2-800 | 79.98 | 2.14 | 1.39 | 16.49 |

**Table S5.** Comparison of 2e⁻ ORR performance between the as-prepared MCPs-0.2 sample in this work and recently reported electrocatalysts.

| **Sample** | | **Electrolytes** | | **Onset potential (V vs. RHE)** | **H_2_O_2_**  **selectivity (%)** | **Refs** |
| --- | --- | --- | --- | --- | --- | --- |
| g-N-CHNs | 0.1 M KOH | | 0.70 | | 63 | 1 |
| B-C | 0.1 M KOH | | 0.77 | | 85 | 2 |
| MCHs | 0.1 M KOH | | 0.83 | | 55 | 3 |
| MCHs-9:1 | 0.1 M KOH | | 0.83 | | 53 | 3 |
| MesoC | 0.1 M KOH | | 0.73 | | 80 | 4 |
| O-CNTs | 0.1 M KOH | | 0.78 | | 90 | 5 |
| BN-C | 0.1 M KOH | | 0.82 | | 90 | 6 |
| O-GOMC | 0.1 M KOH | | 0.81 | | 90 | 7 |
| N,S-TCNTs | 0.1 M KOH | | 0.78 | | 90 | 8 |
| FS-CFs | 0.1 M KOH | | 0.81 | | 85 | 9 |
| Fe–C–O | 0.1 M KOH | | 0.76 | | 95.4 | 10 |
| Co–N–C | 0.1 M KOH | | 0.1 | | 82 | 11 |
| Co_SA_-N-C/CNTs | 0.1 M KOH | | 0.4 | | 85 | 12 |
| **MCPs-0.2** | **0.1 M KOH** | | **0.78** | | **91** | **This work** |

**References:**

[1] D. Iglesias, A. Giuliani, M. Melchionna, S. Marchesan, A. Criado, L. Nasi, M. Bevilacqua, C. Tavagnacco, F. Vizza, M. Prato, P. Fornasiero, *Chem*. **2018**, *4*, 106-123.

[2] Y. Xia, X. Zhao, C. Xia, Z.-Y. Wu, P. Zhu, J. Y. Kim, X. Bai, G. Gao, Y. Hu, J. Zhong, Y. Liu, H. Wang, *Nat. Commun*. **2021**, *12*, 4225.

[3] Y. Pang, K. Wang, H. Xie, Y. Sun, M.-M. Titirici, G.-L. Chai, *ACS Catal.* **2020**, *10*, 7434-7442.

[4] S. Chen, Z. Chen, S. Siahrostami, T. R. Kim, D. Nordlund, D. Sokaras, S. Nowak, J. W. F. To, D. Higgins, R. Sinclair, J. K. Nørskov, T. F. Jaramillo, Z. Bao, *ACS Sustainable Chem. Eng*. **2018**, *6*, 311-317.

[5] Z. Lu, G. Chen, S. Siahrostami, Z. Chen, K. Liu, J. Xie, L. Liao, T. Wu, D. Lin, Y. Liu, T. F. Jaramillo, J. K. Nørskov, Y. Cui, *Nat. Catal*. **2018**, *1*, 156-162.

[6] S. Chen, Z. Chen, S. Siahrostami, D. Higgins, D. Nordlund, D. Sokaras, T. R. Kim, Y. Liu, X. Yan, E. Nilsson, R. Sinclair, J. K. Nørskov, T. F. Jaramillo, Z. Bao, *J. Am. Chem. Soc*. **2018**, *140*, 7851-7859.

[7] J. S. Lim, J. H. Kim, J. Woo, D. S. Baek, K. Ihm, T. J. Shin, Y. J. Sa, S. H. Joo, *Chem.* **2021**, *7*, 3114-3130.

[8] Y. Long, J. Lin, F. Ye, W. Liu, D. Wang, Q. Cheng, R. Paul, D. Cheng, B. Mao, R. Yan, L. Zhao, D. Liu, F. Liu, C. Hu, *Adv. Mater*. **2023**, *35*, 2303905.

[9] F. Xiang, X. Zhao, J. Yang, N. Li, W. Gong, Y. Liu, A. Burguete-Lopez, Y. Li, X. Niu, A. Fratalocchi, *Adv. Mater*. **2023**, *35*, 2208533.

[10] K. Jiang, S. Back, A. J. Akey, C. Xia, Y. Hu, W. Liang, D. Schaak, E. Stavitski, J. K. Nørskov, S. Siahrostami, H. Wang, *Nat. Commun*. **2019**, *10*, 3997.

[11] E. Jung, H. Shin, B.-H. Lee, V. Efremov, S. Lee, H. S. Lee, J. Kim, W. Hooch Antink, S. Park, K.-S. Lee, S.-P. Cho, J. S. Yoo, Y.-E. Sung, T. Hyeon, *Nat. Mater*. **2020**, *19*, 436-442.

[12] H. Hu, C. Zhang, W. Liu, H. Qi, H. Wang, X. Wang, L. Zhang, L. Liu, L. Bao, M. Alomar, J. Zhang, X. Lu, *Adv. Funct. Mater*. **2024**, *35*, 2419220.
